# Targeting the Cell Stress Response of *Plasmodium falciparum* to Overcome Artemisinin Resistance

**DOI:** 10.1371/journal.pbio.1002132

**Published:** 2015-04-22

**Authors:** Con Dogovski, Stanley C. Xie, Gaetan Burgio, Jess Bridgford, Sachel Mok, James M. McCaw, Kesinee Chotivanich, Shannon Kenny, Nina Gnädig, Judith Straimer, Zbynek Bozdech, David A. Fidock, Julie A. Simpson, Arjen M. Dondorp, Simon Foote, Nectarios Klonis, Leann Tilley

**Affiliations:** 1 Department of Biochemistry and Molecular Biology and ARC Centre of Excellence for Coherent X-ray Science, Bio21 Molecular Science and Biotechnology Institute, The University of Melbourne, Melbourne, Victoria, Australia; 2 John Curtin School of Medical Research, the Australian National University, Canberra, Australian Capital Territory, Australia; 3 Australian School of Advanced Medicine, Macquarie University, Sydney, New South Wales, Australia; 4 School of Biological Sciences, Nanyang Technological University, Singapore; 5 Centre for Epidemiology and Biostatistics, Melbourne School of Population and Global Health, University of Melbourne, Parkville, Victoria, Australia; 6 Murdoch Childrens Research Institute, Royal Childrens Hospital, Victoria, Australia; 7 Faculty of Tropical Medicine, Mahidol University, Bangkok, Thailand; 8 Department of Microbiology and Immunology, Columbia University Medical Center, New York, New York, United States of America; 9 Division of Infectious Diseases, Department of Medicine, Columbia University Medical Center, New York, New York, United States of America; 10 Centre for Tropical Medicine, Nuffield Department of Clinical Medicine, Oxford, United Kingdom; Stanford University, UNITED STATES

## Abstract

Successful control of falciparum malaria depends greatly on treatment with artemisinin combination therapies. Thus, reports that resistance to artemisinins (ARTs) has emerged, and that the prevalence of this resistance is increasing, are alarming. ART resistance has recently been linked to mutations in the K13 propeller protein. We undertook a detailed kinetic analysis of the drug responses of K13 wild-type and mutant isolates of *Plasmodium falciparum* sourced from a region in Cambodia (Pailin). We demonstrate that ART treatment induces growth retardation and an accumulation of ubiquitinated proteins, indicative of a cellular stress response that engages the ubiquitin/proteasome system. We show that resistant parasites exhibit lower levels of ubiquitinated proteins and delayed onset of cell death, indicating an enhanced cell stress response. We found that the stress response can be targeted by inhibiting the proteasome. Accordingly, clinically used proteasome inhibitors strongly synergize ART activity against both sensitive and resistant parasites, including isogenic lines expressing mutant or wild-type K13. Synergy is also observed against *Plasmodium berghei* in vivo. We developed a detailed model of parasite responses that enables us to infer, for the first time, in vivo parasite clearance profiles from in vitro assessments of ART sensitivity. We provide evidence that the clinical marker of resistance (delayed parasite clearance) is an indirect measure of drug efficacy because of the persistence of unviable parasites with unchanged morphology in the circulation, and we suggest alternative approaches for the direct measurement of viability. Our model predicts that extending current three-day ART treatment courses to four days, or splitting the doses, will efficiently clear resistant parasite infections. This work provides a rationale for improving the detection of ART resistance in the field and for treatment strategies that can be employed in areas with ART resistance.

## Abstract 2

Resistance to artemisinin antimalarial drugs is jeopardizing malaria control. This study shows that proteasome-mediated stress responses can be targeted to overcome artemisinin resistance and suggests alternate therapeutic regimens and monitoring strategies.

## Author Summary

Resistance to artemisinin antimalarials, some of the most effective antimalarial drugs, has emerged in Southeast Asia, jeopardizing malaria control. We have undertaken a detailed study of artemisinin-sensitive and-resistant strains of *Plasmodium falciparum*, the parasite responsible for malaria, taken directly from the field in a region where resistance is developing. We compared these strains to lab strains engineered with either mutant or wild-type resistance alleles. We demonstrate that in sensitive *P*. *falciparum*, artemisinin induces growth retardation and accumulation of ubiquitinated proteins, indicating that the drugs activate the cellular stress response. Resistant parasites, on the other hand, exhibit reduced protein ubiquitination and delayed onset of cell death following drug exposure. We show that proteasome inhibitors strongly synergize artemisinin activity, offering a means of overcoming artemisinin resistance. We have developed a detailed model of parasite responses and have modelled in vivo clearance profiles. Our data indicate that extending artemisinin treatment from the standard three-day treatment to a four-day treatment will clear resistant parasites, thus preserving the use of this critical therapy in areas experiencing artemisinin resistance.

## Introduction

Malaria remains a scourge of humanity, affecting hundreds of millions of people and causing ~600,000 deaths each year [1]. Infection with *Plasmodium falciparum* is responsible for the majority of severe malaria cases. During the asexual blood phase of its lifecycle, this protozoan parasite invades, grows, and multiplies within red blood cells (RBCs). The initial stage of intraerythrocytic growth (0–~24 h), during which the parasite exhibits an unfilled cytoplasm in Giemsa-stained smears (referred to as “rings”), is characterized by a relatively slow metabolism [2]. Ring-stage—infected RBCs are freely circulating and are thus the predominant stage detected in samples taken from the peripheral blood of infected patients. From ~24 h to ~40 h post-invasion (p.i.), in the “trophozoite” (or growing) stage, the parasite increases the rate of uptake and digestion of hemoglobin from the host cytoplasm and shows a large increase in metabolic rate. These mature parasites are characterized by the presence of hemozoin, the classic “malaria pigment” that results from hemoglobin digestion. Trophozoites are rarely observed in the circulation of infected patients because of their adherence to endothelial cells and consequent sequestration away from the circulation. Complications associated with cerebral sequestration are responsible for much of the malaria-related mortality and morbidity [3]. From ~40 h p.i., the parasite undergoes cytokinesis, forming a schizont that can contain up to 32 daughter parasites (merozoites). At ~48 h p.i., the schizont bursts, releasing the merozoites and heralding a new round of infection.

Artemisinin and its derivatives (collectively referred to as ARTs) have contributed enormously to decreasing rates of malaria deaths over the last decade. ARTs are among the few antimalarials that are active against ring-stage parasites, thus reducing the parasite burden in *P*. *falciparum* infections quickly and providing prompt therapy for severe infections [3]. The ARTs contain an endoperoxide group that is critical for their activity. The mechanism of ART action remains poorly understood, but ARTs are thought to be pro-drugs that need to be activated by opening of the endoperoxide ring, i.e., splitting the bonded oxygen atoms [4]. This process requires the presence of heme or non-heme iron sources (and possibly other activators) [5,6]. The activated ART intermediates are thought to react with susceptible (nucleophilic) groups within parasite proteins and other cellular components, leading to parasite killing; however, the details remain unclear [7].

A disadvantage of ARTs is their short half-lives in vivo (~1–2 h). Accordingly, they are co-administered with longer half-life partner drugs in ART combination therapies (ACTs) to prevent recrudescence and to slow the emergence of resistance [8]. Current antimalarial control is highly dependent on ACTs, which makes the emergence of ART resistance extremely concerning [9–11]. Decreased sensitivity to ARTs, which manifests as delayed parasite clearance, is now a problem in six Southeast Asian countries and is translating into decreased clinical efficacy in areas with concomitant partner-drug resistance [12,13]. Enormous efforts are underway to contain and eliminate ART resistance.

Initially, monitoring ART resistance was hampered by the lack of a suitable in vitro correlate [10]. Recently, assays employing short pulses mimicking clinical drug exposure [14–16], such as the ring-stage survival assay (RSA), have provided a good correlation between reduced in vitro sensitivity to dihydroartemisinin (DHA), the clinically relevant ART derivative, and delayed parasite clearance [15]. Combined with whole genome sequencing, this allowed the identification of mutations in a protein with a Kelch domain (i.e., β-propeller tertiary structure, referred to as K13; PF3D7_1343700) [17] that are strongly associated with the slow-clearance phenotype. A large Genome-Wide Association Study (GWAS) added further support to the suggestion that *K13* is the major locus controlling *P*. *falciparum* resistance to ARTs [18]. Recent studies using genetic modification of the *K13* locus have confirmed a central role for K13 mutations in conferring ART resistance [19,20].

Here we present a detailed in vitro investigation of the drug responses of K13 wild-type and mutant isolates of *P*. *falciparum* sourced from a region in Cambodia (Pailin) with a marked penetrance of ART resistance [10,21]. Our results provide insights into the molecular basis of ART action and resistance and point to a class of compounds that could be used to synergize the activity of ARTs against both sensitive and resistant parasites. Modelling of parasite responses suggests alternate therapeutic regimens that should be vigorously pursued and provides tools that will immediately impact the way resistance is measured in the field.

## Results

### Whole Genome Sequencing of K13 Wild-Type and Mutant Strains from Pailin

We determined the whole genome sequences of four laboratory-adapted isolates of *P*. *falciparum* collected from adult patients enrolled in clinical trials conducted between 2009 and 2010 in the Pailin Referral Hospital in Cambodia [21]. High sequencing coverage with good mapping quality was achieved across all four genomes (mean sequence coverage of 77x and mean mapping quality >30; see S1 Table). A Pairwise Distance matrix (5x5) built on 26,438 SNPs in coding genes was used to generate a Neighbor Joining tree to examine isolate relatedness. The isolates are quite divergent, with ~16,000 non-synonymous SNPs between any two isolates (excluding var, rif, and stevors), and they exhibit multiple mutations in many of the drug-resistance—related genes.

One strain (PL2) encodes the wild-type K13 genotype, while the others (PL1, Y493H; PL5, C580Y and PL7, R539T) represent three K13 mutants that are commonly observed in Cambodia (S2 Table). A recent fine-structure analysis of parasite samples collected as part of the large-scale Tracking Resistance to Artemisinin Collaboration (TRAC) study revealed that particular non-synonymous polymorphisms in apicoplast ribosomal protein S10 (*arps10*), multidrug resistance protein 2 (*mdr2*), ferredoxin (*fd*), and chloroquine resistance transporter (*pfcrt*) are markers of a genetic background on which *K13* mutations are likely to arise, but individually they have little contribution to ART resistance [18,22]. Other GWAS have reported SNPs in other genes that show association with the ART resistance phenotype [23–27]. The K13 mutant and wild-type Pailin strains exhibited the expected SNPs at the *arps10* (V127M) and *mdr2* (T484I) loci and at one of the *pfcrt* (I356T) loci but showed variable sequences at the other loci (see S2 Table for a summary of some relevant loci). Both K13 mutant and wild-type strains exhibited the *fd* (D193Y) and *pfcrt* (N326S) SNPs that are strongly associated with ART resistance founder populations [18]. The entire genomes for the four Pailin strains have been made available through the European Nucleotide Archive (ENA) database under the accession number PRJEB8074.

### K13 Mutants Exhibit Decreased ART Sensitivity during One-Third of the Blood Cycle

We previously demonstrated that the sensitivity of laboratory strains of *P*. *falciparum* to ARTs exhibits a complex dependence on drug exposure time and concentration [14]. For this work, we used assays comprising very short drug pulses in an effort to mimic in vivo exposure and to maximize the discrimination of subtle differences in parasite responses. We defined parasite viability as the fraction of the parasite population that survives drug exposure and is able to enter the next parasite cycle. We found that the 50% lethal dose (*LD*_*50*_) and the viability at saturating drug concentrations (the minimum viability, *V*_*min*_) are the most useful measures of cellular cytotoxicity, in agreement with other studies of ARTs and other drugs [15,28,29].

**Fig 1:**
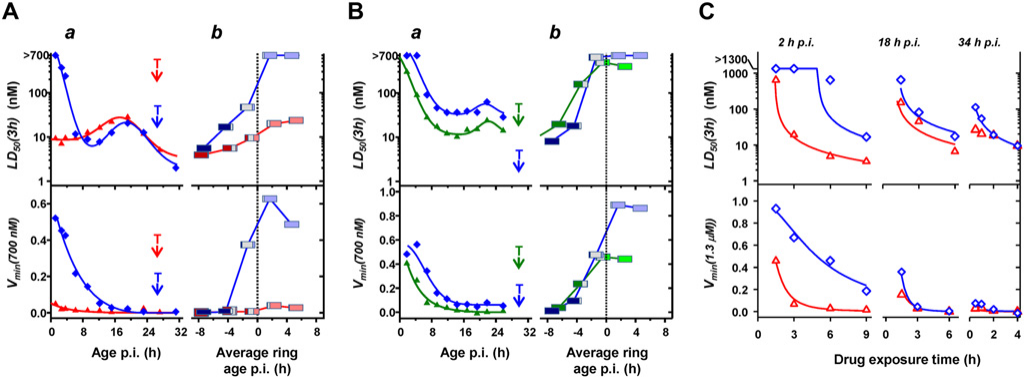
Stage-dependent in vitro DHA responses of Pailin strains. (A,B) Tightly synchronized PL2 and PL7 (A, red and blue, respectively) and PL1 and PL5 (B, green and blue, respectively) cultures were assayed using 3-h DHA pulses across the blood cycle. The *a* panels show the variation of *LD*_*50*_ *(3h)* and *V*_*min*_ *(3h)* during the first 32 h p.i. Time points corresponding to 50% trophozoites (T; filled cytoplasm in Giemsa stained smear) are indicated. The *b* panels show the variation of *LD*_*50*_ *(3h)* and *V*_*min*_ *(3h)* across the schizont-to-ring transition. Post-invasion ages are normalized to those in the next cycle to account for the different lifecycle durations of the different strains (49, 52, 59, and 57 h for PL1, 2, 5, and 7, respectively). Negative ages represent pre-invasion ages and correspond to schizonts that are the indicated time from forming new rings. The midpoint of each box corresponds to the average parasite age at the start of each assay, normalized to a value of zero when 50% of the parasites had formed rings. Colored bar widths reflect the relative parasite populations at the beginning of each assay: schizonts (dark blue for PL7 and PL5, dark red for PL2, and dark green for PL1), rings (light blue for PL7 and PL5, light red for PL2, and light green for PL1), and schizonts forming rings during the course of the assay (grey for all strains). (C) Tightly synchronized PL2 (red) and PL7 (blue) cultures were subjected to DHA pulses of different durations at 2, 18, and 34 h p.i. Curves represent best fits obtained with the cumulative effective dose (CED) model, with the parameters shown in Table 1. Dose response profiles for each exposure time are shown in S1 Fig.

In the current work, tightly synchronized cultures of the four strains were subjected to 3-h pulses of DHA at different stages of the asexual lifecycle (Fig 1A and 1B). Notably, the PL2 strain exhibits a low *V*_*min*_ *(3h)* (<5%) across all stages of development (Fig 1A), confirming an ART sensitive phenotype. In contrast, the PL1, 5, and 7 strains displayed 5%–60% survival over the first 10–12 h p.i. (Fig 1A and 1B). The youngest ring stages (1.2 h p.i.) of the mutant strains exhibited the greatest viability following drug exposure (*V*_*min*_ *(3h)* >40%), as previously reported [15], confirming the resistant phenotype of these strains. Of note, the PL2 and PL7 strains exhibit similar *LD*_*50*_ *(3h)* values at 6 h p.i., but PL7 shows a higher capacity to survive drug exposure (*V*_*min*_ *(3h)* = 20%) (Fig 1A). Thus *V*_*min*_ *(3h)* appears to be the more sensitive indicator of the resistance genotype, as suggested previously [14].

We also monitored the drug response of mature parasites during their transition into the next cycle (Fig 1A and 1B, *b* panels). Continuous in vitro culturing of parasite cultures results in broadening of the age distribution over time. We measured the degree of broadening in this study (e.g., the schizont-to-ring transition shown in the *b* panels in Fig 1). In agreement with a previous study [14], ~80% of parasites that are synchronized to a 1-h window undergo the schizont-to-ring transition over a period of ~4 h in the next cycle (i.e., ~48 h later). Late trophozoites and early schizonts (average ages earlier than 6 h pre-invasion) of all strains exhibit similar *LD*_*50*_ *(3h)* values with no detectable survival when exposed to 0.7 μM DHA. By contrast late-stage PL1, 5, and 7 schizonts (average ages later than 4 h pre-invasion), which form rings during the course of the assay, exhibit increased *LD*_*50*_ *(3h)* and *V*_*min*_ *(3h)* values. This indicates that the mutant strains have an increased ability to survive a clinically relevant drug pulse (0.7 μM DHA; 3 h) from -4 to +12 h p.i., encompassing almost one-third of the intraerythrocytic cycle. The ability of late-stage schizonts to survive exposure is particularly important, considering each surviving parasite forms approximately ten daughter cells.

There are subtle differences between the responses of the different K13 mutants. These may be due to additional genetic factors as well as to direct effects of the different mutations, as indicated by a detailed reverse genetics analysis of the contribution of K13 to resistance in recent Cambodian isolates and reference lines [20]. The time points for transition from ring to trophozoites (i.e., the 50:50 point; indicated with "T" in Fig 1A and 1B) and the lifecycle durations for the K13 mutant and wild-type parasites were: PL1 (30 h/49 h), PL5 (30 h/59 h), PL7 (27 h/57 h) and PL2 (27 h/52 h), and 3D7 (22 h/41 h). Thus, among this small sample size, there does not appear to be a simple correlation between the K13 mutation and the length of the ring stage or of the intraerythrocytic cycle.

### The Drug Responses of K13 Mutant and Wild-Type *P*. *falciparum* Can Be Modelled in Terms of a Cumulative Effective Dose

Our previous work with laboratory strains showed that ART-mediated killing requires that parasites are exposed to a sufficient concentration of activated ART for a sufficient period of time [14]. We defined a semi-empirical cumulative effective dose (CED) model that accounts for the complex in vitro dependence of parasite viability on drug concentration and exposure time and permits the interpretation of stage and strain-dependent differences in drug action in terms of easy to understand underlying parameters. Here we analyzed the dependence of DHA action on time of exposure in K13 wild-type (PL2) and K13 mutant (PL7, R539T) field strains (Figs 1C and S1).

A 3-h exposure of PL2 (2 h p.i.) to 1.3 μM DHA is sufficient to reduce parasite viability to almost zero (Fig 1C, red). Remarkably, a significant fraction (20%) of PL7 early rings (Fig 1C, blue) remain refractory to a 9-h exposure to 1.3 μM DHA, even though the *LD*_*50*_ *(9h)* value (20 nM) suggests potent drug action at longer exposures; this further illustrates that *V*_*min*_ is more informative than *LD*_*50*_ in revealing the resistance-associated phenotype. Late rings (18 h p.i) and trophozoites (34 h p.i.) from both strains show similar sensitivity to >3 h exposure to DHA, but small differences in *LD*_*50*_ are evident at very short exposure times (Fig 1C).

We found that the CED model is able to adequately describe the response of the Cambodian strains to DHA at different stages of development, as well as the dependence of that response on concentration and exposure time. That is, the CED model can be used to generate the fitted curves in Fig 1C and S1 Fig at the very early ring (2 h p.i.), the late ring (18 h p.i.) and the mid trophozoite (34 h p.i.) stages. In this model, the effective dose (ED) is a saturable function of the drug concentration and is defined by *K*_*m*_, the drug concentration resulting in half the effect of the maximally effective dose, *ED* ^*max*^. Parasite viability is then a sigmoidal function of the cumulative ED (*ED* ^*cum*^) with slope *γ* and midpoint $t_{50}^{e,sat}$. The $t_{50}^{e,sat}$ value is the time taken to kill half of the parasites at saturated (and fixed) drug concentration. (See [14] for further explanation of the model).

The drug response of late rings and mid trophozoites was similar for both wild-type and mutant strains and the analysis indicated similar CED model parameters (Table 1; Fig 1C). Interestingly, early rings from the K13 wild-type and mutant strains, which produce quite a different drug response, exhibit similar *K*_*m*_ values (14–19 nM; Table 1). This suggests that DHA initiates an effect in both drug-sensitive and drug-resistant strains at a similar concentration, even in the early ring stages. Since the ARTs are pro-drugs that are converted to the active form by reaction with iron or heme [4,6], this suggests that all strains activate the drug with similar efficiency. By contrast, there is a 3.5-fold increase in the $t_{50}^{e,sat}$ values for DHA for early ring stage PL7 parasites (Table 1), and this underlies most of the difference in the response of this strain. As previously demonstrated for laboratory strains [14], such an increase in $t_{50}^{e,sat}$ manifests as an increase in the lag time for drug action. In other words, there is a longer delay following drug treatment before the onset of the killing of the K13 mutants. Since $t_{50}^{e,sat}=ED_{50}^{cum}/ED^{\max}$ (where $ED_{50}^{cum}$ is the cumulative ED required to kill 50% of the parasites [14]), this indicates that PL7 requires longer exposure to an effective dose to induce killing (or the maximal effective dose produced is less). This difference is expected to be very important in vivo given the very short in vivo half-lives of ARTs.

**Table 1:** CED model parameters for PL2 and PL7 strains.

Parameter	Parasite Strain and Stage					
	Ring (2 h p.i.)		Ring (18 h p.i.)		Troph (34 h p.i.)	
	PL2	PL7	PL2	PL7	PL2	PL7
*γ*	2.6	1.9	2.6	3.6	ND	2
$t_{50}^{e,sat}$(h)	1.4	4.9	0.9	1.2	ND	0.2
*K*_*m*_ (nM)	19	14	60	88	ND	16

ND: parameters could not be reliably determined

### K13 Mutant and Wild-Type Parasites Exhibit Similar DHA-Mediated Cytostatic Effects That Precede Killing

A number of studies have suggested that ARTs can exert cytostatic effects at sub-lethal concentrations [30,31]. Here we have used the RNA-binding dye SYTO-61 (which can readily distinguish parasites of different ages [30]) to determine whether the parasites that survive DHA exposure exhibit growth retardation. (See Materials and Methods for labelling strategy).

**Fig 2:**
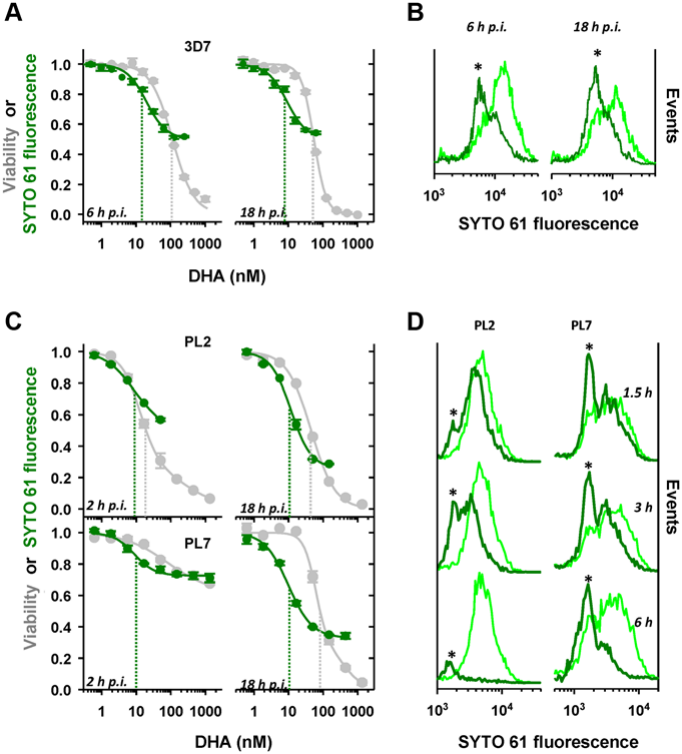
DHA induces growth retardation prior to killing. (A, C) DHA-induced decrease in the SYTO-61 signal of viable parasites. Tightly synchronized 3D7 (A) and PL2 and PL7 (C) parasites were subjected to 4-h (A) or 3-h (C) DHA pulses at the ring-stage ages indicated in the figure. The median SYTO-61 signals of viable parasites were determined in the next cycle (see Materials and Methods). The concentration dependence of the SYTO-61 signal (growth effects; green symbols) is compared with that of parasite viability (grey symbols). Dashed green and grey lines represent the *IC*_*50*_ and *LD*_*50*_ values associated with each exposure time. Error bars correspond to the range of duplicates. (B,D) Changes in the SYTO-61 staining profile of viable parasites after drug exposure. 3D7 parasite (6 and 18 h p.i., B) were subjected to 4-h DHA pulses at 63 and 16 nM, respectively. Alternatively PL2 and PL7 late rings (18 h p.i.) were subjected to 1.5, 3, and 6-h DHA pulses (16 nM; D). The SYTO-61 signals were measured by flow cytometry in the next cycle. The SYTO-61 distributions of the viable population (dark green) are compared with those of untreated parasites (light green). Asterisks highlight an absolute increase in the number of younger parasites, indicating growth retardation.

We initially examined growth effects in the laboratory strain, 3D7, at the ring stage of development, where it exhibits 10-fold lower sensitivity to a 3-h DHA pulse compared to the trophozoite stage [14]. We found that mid-ring (6 h p.i.) and later ring (18 h p.i.) stage parasites that survive exposure to a 4-h drug pulse exhibit a dose-dependent decrease in the intensity of the SYTO-61 signal following treatment (Fig 2A, green curves). The *IC*_*50*_ *(4h)* for the growth retardation effect was similar across the ring stage (~10 nM) and was 5- to 10-fold lower than the corresponding *LD*_*50*_ *(4h)* value (i.e., cytotoxic effect). Examination of the population profile of SYTO-61 staining shows an absolute increase in the number of viable parasites with decreased SYTO-61 signals in the drug-treated samples compared to the untreated controls (Fig 2B, asterisks). This indicates that the decrease in the SYTO-61 signal following drug exposure reflects drug-induced growth retardation rather than selective killing of the oldest parasites.

Similarly for both the K13 wild-type (PL2) and R539T mutant (PL7) strains, early ring-stage parasites (2 h p.i.) that survive drug exposure exhibit dose-dependent growth retardation (Fig 2C, green curves). Interestingly, the two strains exhibit similar *IC*_*50*_ *(3h)* values for growth retardation (10 nM) despite their very different sensitivities to killing (*LD*_*50*_ *(3h)* = 10 and >1000 nM for PL2 and PL7, respectively; Fig 2C, compare green and grey curves). These *IC*_*50*_ *(3h)* values remain relatively constant throughout the ring-stage, with the maximum effect evident in late rings (Fig 2C, right panels).

**Fig 3:**
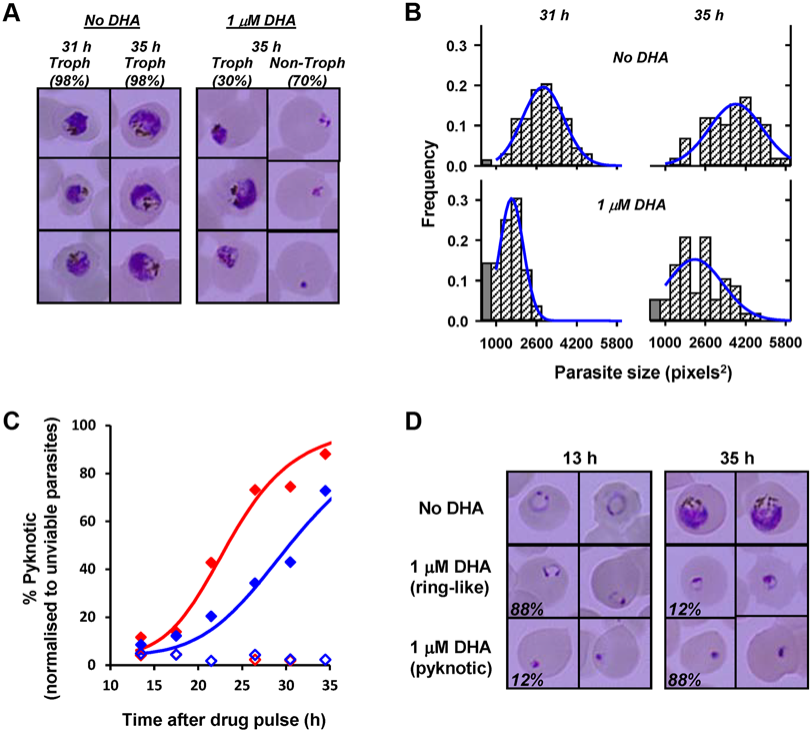
Quantitation of growth retardation and transition to pyknotic forms following DHA treatment. (A) Early ring-stage PL7 parasites (1.5 h p.i.) were treated with a 3.5 h DHA pulse (1 μM) and the morphology of parasites (Giemsa smears) examined at 31 and 35 h after the drug pulse. Representative images of untreated and treated parasites are shown. The proportion of the parasite population corresponding to trophozoites (hemozoin-containing parasites) and non-trophozoites (rings or pyknotic forms) are indicated in the figure. (B) The size distribution of viable parasites (hemozoin-containing) in the drug-treated culture was quantitated and compared with untreated cultures at the indicated times after the drug pulse (*n* >50 per sample). Late rings are larger than early trophozoites and were counted as a separate category (filled grey bars). (C) PL2 (red) and PL7 (blue) parasites (1.5 h p.i.) were rendered unviable by treatment with a DHA pulse (1 μM, 3.5 h) and the fraction of unviable parasites exhibiting pyknotic morphology was quantitated (filled symbols). Open symbols denote untreated parasites. (D) Representative images of untreated PL2 parasites and treated unviable parasites showing ring-like and pyknotic morphologies (fraction of each morphology indicated).

We compared the effect of the time of exposure to drug on growth retardation and viability. The response of the PL2 and PL7 strains at late-ring stage to exposure to DHA for a very short period (1.5 h) is similar, and mainly comprises growth effects without loss of viability (Fig 2D). Differences in viability manifest at longer exposures (Fig 2D, 3 and 6h). While PL2 parasites succumb to longer drug exposure, the growth-retarded PL7 parasites are able to withstand drug pressure for longer. This demonstrates that it is the ability of the growth-retarded PL7 parasites to withstand subsequent drug pressure that is responsible for their resistance phenotype.

Drug-induced growth retardation effects are important as they will influence the stage at which surviving parasites are exposed to recommended ART regimens, which often include daily doses administered 24 h apart. We quantitated the magnitude of this growth effect by subjecting tightly synchronized PL7 parasites (1.5 h p.i.) to a 3.5-h DHA pulse (1 μM) and periodically examining parasite morphology by Giemsa staining over a period of 40 h, following the drug pulse. At times >30 h, two distinct parasite populations were identified corresponding to those containing hemozoin (predominantly trophozoite morphologies) and those with pyknotic or early ring morphologies (Fig 3A). The fraction of parasites exhibiting a trophozoite-like appearance (30%; *n* >100 parasites) matched the viability as measured by flow cytometric analysis in the cycle following the drug pulse (28%), indicating that these trophozoites represent the viable population and confirming this as a simple method for measuring loss of viability (i.e., parasites rendered incapable of reproduction) in the same cycle as the drug treatment. A comparison of the sizes (areas of Giemsa-stained parasites) of the surviving trophozoites with those from an untreated culture shows that the surviving parasites were, on average, delayed 6 h in their progression through the cycle and exhibited a broader age distribution (Fig 3A and 3B).

### Unviable K13 Mutant and Wild-Type Parasites Exhibit Differential Rates of Conversion to Pyknotic Forms

The above analysis indicated that unviable parasites (i.e., incapable of reproduction) can exhibit normal ring morphologies for some time after exposure to ART. We examined the timing of the appearance of pyknotic forms in Giemsa smears, following a DHA pulse, to determine whether the differential drug response of sensitive and resistant strains also influenced the rate of generation of pyknotic forms. Early ring-stage parasites (1.5 h p.i.) were pulsed with 1 μM DHA for 3.5 h to generate unviable parasites (100% and 72% of PL2 and PL7 parasites, respectively, based on flow cytometric analysis in the cycle following the drug pulse), and parasite morphology was quantitated (from Giemsa smears) every 2–4 h following the drug pulse. Surprisingly unviable PL2 and PL7 parasites retained a ring-like morphology 13 h after the drug pulse, with pyknotic forms comprising <10% of the unviable population (Fig 3C and 3D). A significant fraction of unviable parasites from both strains (12% and 28% for PL2 and PL7, respectively) exhibited ring-like morphologies 35 h following the drug pulse. Interestingly, the half-time for adoption of pyknotic morphology was significantly longer for PL7 (32 h) than for PL2 (23 h). This indicates that unviable resistant parasites retain a ring-like morphology for longer following DHA treatment. In vivo, these unviable K13 mutant parasites may remain in the blood stream for longer.

### Cytostatic effects reflect a cellular stress response that engages the ubiquitin-proteasome pathway

The growth retardation caused by exposure to DHA is reminiscent of the cell stress response observed in other organisms. In other systems, cellular insults can result in protein unfolding, culminating in polyubiquitination of proteins and their destruction via the proteasome. Interestingly, a very recent analysis of the transcriptomes of parasite samples collected as part of the TRAC study provided evidence for up-regulation of protein homeostasis genes (such as the ubiquitin-proteasome pathways) that correlates with delayed clinical clearance of parasites [32]. To determine the level of protein damage following ART treatment, we sought to determine the level of ubiquitinated proteins in parasite extracts.

We found that we were not able to reliably measure the parasite-associated signal above the host RBC background at the very early ring stage (mean fold change = 0.9 ± 0.2; n = 5). Therefore we examined effects in trophozoites (which have much higher levels of protein ubiquitination) where K13 wild-type and mutant trophozoite stage parasites show less dramatic, but still measureable differences in response to very short pulses of DHA (see Fig 1C, 34 h p.i). We found that this differential response was more pronounced when parasites were treated with the less potent parent drug, artemisinin (qinghaosu; QHS). We found that a very short pulse (90 min) of QHS killed PL7 much less efficiently than PL2 and 3D7 (*LD*_*50*_ *(1*.*5h)* values of 857, 208, and 153 nM, respectively.

**Fig 4:**
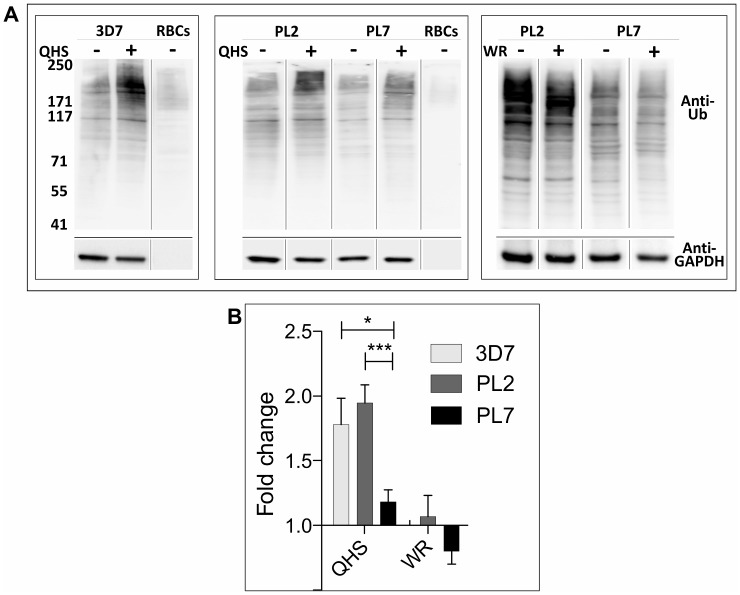
Ubiquitination of *P*. *falciparum* proteins following ART treatment. Uninfected RBCs or trophozoite-infected RBCs (24–44 h p.i.) (3% hematocrit) of 3D7, PL2, and PL7 strains were incubated with 1 μM QHS or 20 nM WR99210 for 90 min at 37°C. Cell extracts were subjected to SDS-PAGE and western blotting and probed with anti-ubiquitin IgG with ECL detection, then stripped and re-probed with anti-*Pf*GAPDH. (A) Representative western blots. (B) Densitometric analysis of the anti-ubiquitin signal for at least nine QHS and three WR99210 experiments. Significance was determined using a Student’s *t* test. * *p* <0.05; *** *p* <0.005.

To determine the effect of QHS treatment on cell stress levels, infected RBCs were saponin-lysed to release the soluble RBC cell contents and parasite extracts were subjected to SDS-PAGE and probed with an antibody that recognizes ubiquitin (Fig 4A). A profile of ubiquitination was observed similar to that reported previously [33], with protein ubiquitination in trophozoites notably higher than in uninfected RBC ghosts. The level of protein ubiquitination increased significantly upon ART treatment (90 min pulse of 1 μM QHS), consistent with engagement of the ubiquitin-proteasome system. The level of protein ubiquitination was higher in ART-treated 3D7 and PL2 parasites than in PL7 parasites (Fig 4A), consistent with the resistant parasites experiencing a lower level of cellular stress. By contrast, a 90 min pulse treatment with 20 nM WR99210 (a concentration sufficient to cause 100% killing) had no effect on the level of ubiquitination (Fig 4A, right panel). A quantitative analysis of several experiments is presented in Fig 4B.

### Cellular Stress Pathways Can Be Targeted to Enhance ART Activity against Both Sensitive and Resistant Parasites

**Fig 5:**
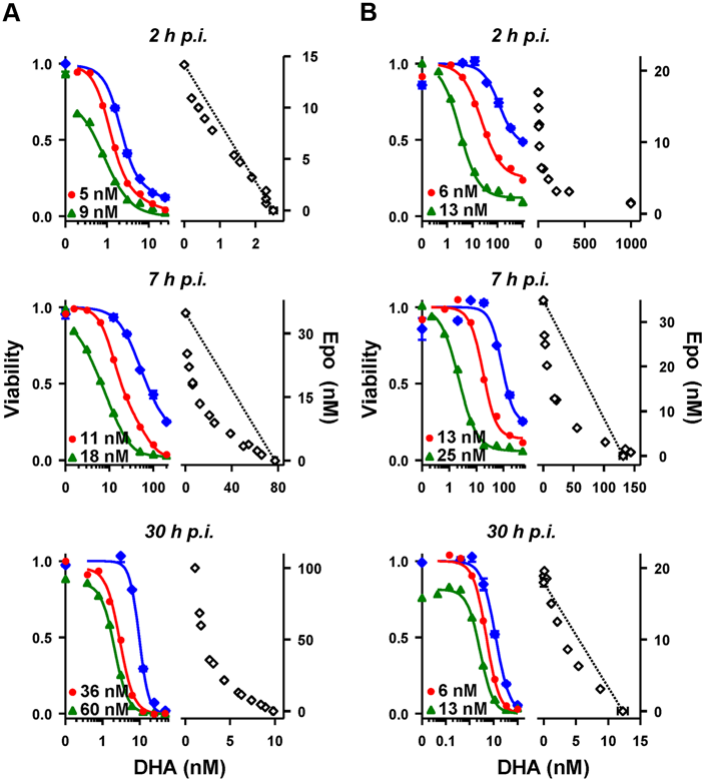
Synergy of DHA and a proteasome inhibitor against *P*. *falciparum*. The interaction of DHA and epoxomicin (Epo) was examined using (A) 3D7 and (B) PL1 parasites at the ages indicated. Left panels in (A and B) show the dose-responses to DHA (blue symbols) and the influence of sublethal concentrations (indicated) of epoxomicin. Right panels in (A and B) show isobolograms for the epoxomicin-DHA pair at the 50% *LD*_*50*_ *(3h)* level. The dashed line is plotted between the *LD*_*50*_ *(3h)* of each drug when used alone, emphasizing the concave nature of many of the isobolograms. The absence of a dashed line indicates the *LD*_*50*_ *(3h)* value of one of the drugs was outside the range of concentrations examined. Error bars, where present, correspond to the range of duplicates.

Given the evidence for accumulation of ubiquitinated proteins following ART treatment, we examined the effects of inhibitors of the proteasome; a proteinase complex that plays a critical role in degrading unfolded proteins. Proteasome complexes are present in both the host and parasite cytoplasm, though selective inhibition of the host proteasome does not affect parasite growth or replication [34,35]. Epoxomicin is a well-characterized and highly specific proteasome inhibitor [36] and has activity against the *P*. *falciparum* proteasome [37]. Epoxomicin showed activity against all stages of the field strains, when used alone, with maximal potency against early ring-stages (see *y*-axis intercepts in right panels in Figs 5 and S2). This activity was independent of *K13* genotype.

We examined the interaction between DHA and epoxomicin by examining the effect of epoxomicin on the dose response profile of DHA, and by measuring the isobologram for the pair of drugs [38] at the 50% lethal dose level. We initially examined 3D7 parasites. Unlike the Pailin strains, early ring-stage 3D7 parasites (2 h p.i.) exhibit ART hypersensitivity [14] and we observed no interaction between DHA and epoxomicin at this stage (Fig 5A, top) panel. In contrast, a sub-lethal concentration of epoxomicin (18 nM) enhances the potency of DHA ~10-fold against the ring-stage of 3D7 (Fig 5A, middle panel); this is illustrated by the concave shape of the *LD*_*50*_ isobologram. This suggests that the proteasome inhibitor overcomes the cell defense systems that protect the mid-ring stage of 3D7.

We next examined the ability of epoxomicin to synergize the action of DHA against the K13 mutant isolate, PL1. A pronounced synergistic interaction is evident at early ring and ring stages (Fig 5B). Notably, some synergism is also evident in the trophozoite stage (Fig 5B, bottom panel). This is in stark contrast to the effect of hemoglobinase inhibitors, which produce strong antagonistic interactions in the trophozoite stage [30]. We observed a similar synergistic interaction with two other K13 mutant strains (PL5 and 7), with pronounced synergism at early-ring and ring stages (S2B and S2C Fig). The synergistic effect was less pronounced but still evident in the Pailin K13 wild-type isolate (PL2) (S2A Fig).

Very recently, modification of the *P*. *falciparum K13* locus in defined genetic backgrounds was used to demonstrate a central role for K13 mutations in conferring ART resistance [20]. These studies included a laboratory-adapted K13 mutant isolate from Cambodia (Cam3.II_ R539T) and a reverted transfectant in the same line, in which the *K13* wild-type genotype has been restored. We examined the sensitivity of these parasites to DHA at different stages of intraerythrocytic development (S3 Fig). Cam3.II exhibited marked resistance to DHA in the very early ring-stage with a *V*_*min*_ *(3h*, *1 μM)* value of 69% and an *LD*_*50*_ value of >>1 μM (S3 Fig), while the Cam3.II_rev line showed markedly enhanced sensitivity with a *V*_*min*_ *(3h*, *1 μM)* value of 8% and an *LD*_*50*_ value of 47 nM, in good agreement with the recent report [20]. We examined the interaction of epoxomicin with DHA against Cam3.II_R539T and Cam3.II_rev. Epoxomicin exhibited very strong synergism with DHA in the very early ring-stage of the Cam3.II_R539T isolate (S3 Fig), consistent with the proteasome inhibitor overcoming the K13-mediated resistance mechanism. However, synergism was also observed in the ring and trophozoite stages of Cam3.II and in all stages of the revertant line (S3 Fig). This confirms that proteasome inhibitors can enhance the activity of ARTs against both sensitive and resistant parasites.

Proteasome inhibitors are used clinically in humans to treat myeloma [26]. We examined the effect of two of these compounds, Carfilzomib, an epoxyketone, and Bortezomib, a peptide boronate [39]. Like epoxomicin, Carfilzomib was potent against all strains and stages examined and exhibited strong synergism with DHA, particularly in the less sensitive, very early ring stage (S4 Fig). Similarly, the clinically used proteasome inhibitor Bortezomib exhibited strong synergism with DHA (S5 Fig). These results confirm that the proteasome is involved in the parasite's response to DHA and that inhibiting its activity enhances the level of killing of the parasite.

### A Proteasome Inhibitor Acts Synergistically with DHA In Vivo

**Fig 6:**
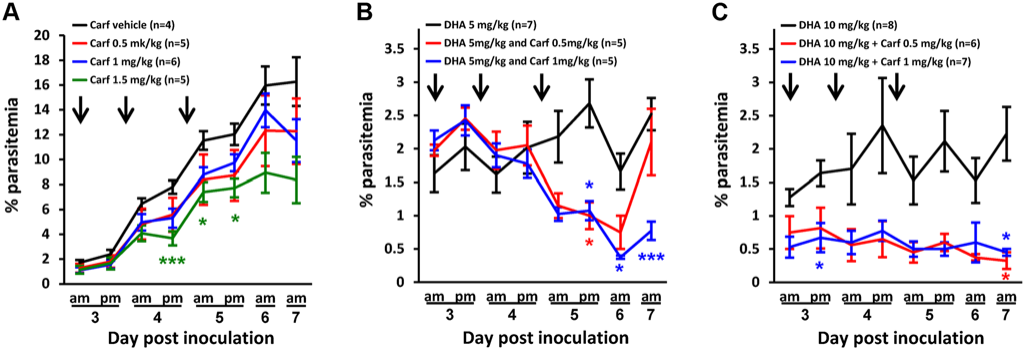
In vivo synergy between DHA and Carfilzomib in a *P*. *berghei* mouse model. Balb/c mice were infected with 10^6^ parasites (*P*. *berghei*) and treatment was initiated at ~1% parasitaemia (i.e., day 3), indicated with an arrow. Data represent averages of data from three independent experiments. (A) Carfilzomib alone has no effect on parasite growth at doses of 0.5 and 1 mg/kg, but has a slight antimalarial effect at 1.5 mg/kg. (B) Treatment with DHA (5 mg/kg) + Carfilzomib (0.5 mg/kg) reduces parasite growth, while DHA (5 mg/kg) + Carfilzomib (1 mg/kg) prevents parasite growth. (C) DHA (10 mg/kg) + Carfilzomib (0.5 or 1 mg/kg) also reduces parasitemia by 2- to 3-fold. Error bars represent SEM and * and *** represent, respectively, *p* <0.05 and *p* <0.01, after Bonferroni correction for multiple testing.

Proteasome inhibitors such as Carfilzomib have previously been tested for in vivo activity against a murine model [40]. While the activity of Carfilzomib alone against *P*. *berghei* is low, we were interested to determine whether sub-lethal concentrations of Carfilzomib might synergize the activity of DHA in the *P*. *berghei* mouse model. In agreement with a previous report [40], we found that treatment with up to 1 mg/kg of Carfilzomib monotherapy had no toxic effects, but also had no beneficial effects in reducing parasite burden (Fig 6A). We found that DHA treatment alone (initiated at ~1% parasitaemia) at a dose of 5 or 10 mg/kg/day gave a moderate decrease in parasite burden (Fig 6B and 6C), while 15 or 20 mg/kg was sufficient to abrogate the parasite burden (S6A and S6B Fig). By contrast, a combination of Carfilzomib (0.5 or 1 mg/kg/day) and DHA (5 mg/kg/day) was associated with a significant reduction in the parasite growth (Fig 6B, red and blue curves) while a combination of Carfilzomib (0.5 or 1 mg/kg) and DHA (10 mg/kg) almost completely abrogated the parasite burden (Fig 6C, red and blue curves). Of particular interest is the observation that a combination of DHA (5 mg/kg) + Carfilzomib (1 mg/kg) (S6C Fig, green curve) or DHA (10 mg/kg) + Carfilzomib (0.5 and 1 mg/kg) (S6C Fig, blue and purple curves) largely abrogated the parasites in the circulating reticulocytes, the blood cell preferentially parasitized by *P*. *berghei*. This further confirms the role of the proteasome in protecting malaria parasites against the toxic effects of DHA and points to a possible means of synergizing the activity of ARTs in vivo, using repositioned proteasome inhibitors.

### Modelling In Vivo Parasite Clearance

The availability of the data from our extensive kinetic analysis of the K13 wild-type and mutant Pailin strains offers the possibility of modelling the drug response of resistant and sensitive parasites at different exposure times and concentrations in vitro and also of extending this analysis to infer behavior in vivo [14]. To do this, we extended the CED mathematical model to take into account the age- and exposure-time—dependence of the drug responses of the sensitive and resistant strains, as well as drug-induced growth retardation and population broadening. We provide an Excel spreadsheet that presents the Mathematical Model in a user-friendly format (S1 Spreadsheet). We have provided a presentation in Prezi that explain the steps involved in using the spreadsheet (https://prezi.com/9xo9b8igvjzl/).

**Fig 7:**
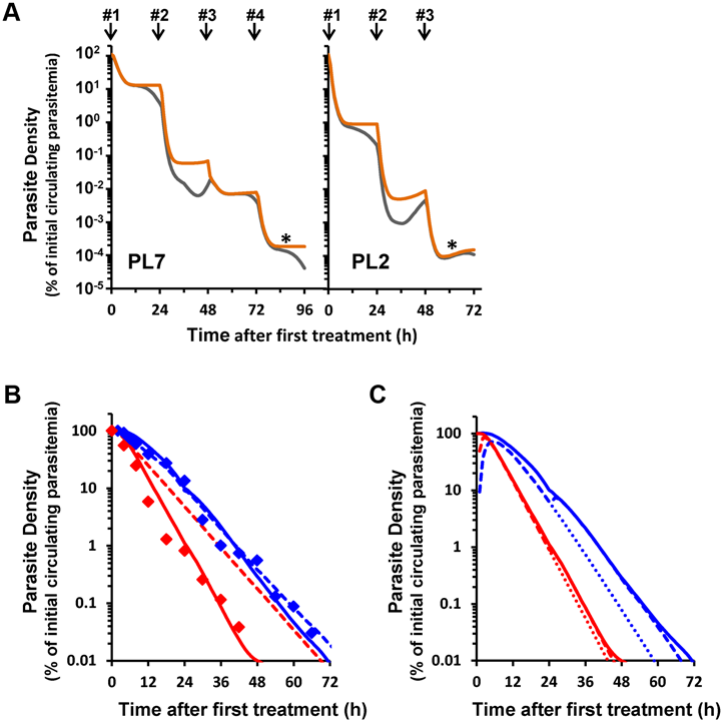
Delayed parasite clearance of resistant strains reflects both reduced killing and delayed removal of unviable parasites. (A) Viable parasite load during DHA treatment regimen. The number of total (circulating and sequestered; orange curves) and circulating (grey curves) viable parasites during DHA therapy in hypothetical patients presenting with ring-stage PL7- and PL2-like infections were simulated using CED model parameters and an in vivo DHA pharmacokinetic profile (see S1 Appendix, S3 Table). Arrows indicate the times of drug administration. (B) Influence of unviable parasite removal on parasite clearance profiles. The circulating unviable parasites in (A) in the PL2 and PL7-like infections (red and blue curves, respectively) were modelled to disappear from circulation with a half-life of 5 h (dashed curves) or exhibit more complex removal (solid curves) involving strain-dependent (1- and 3.5-h half-lives for PL2 and PL7), in addition to a strain-independent (3-h half-life) component. Symbols correspond to median circulating parasite loads from patients in Pailin (blue) and Wang Pha (red) [41]. (C) Contribution of unviable parasites to parasite clearance curves. The solid curves represent the simulated parasite clearance curves shown in Fig 7B incorporating the complex clearance of unviable parasites. The dashed curves correspond to the total number of unviable parasites at each time point and the dotted curves to the unviable parasites produced during the course of the first treatment (i.e., formed during the first 24 h).

We applied the model to predict parasite clearance rates in vivo during a three-day course of ART monotherapy. We assumed DHA is applied at a rate of 2 mg/kg at 0, 24, and 48 h, i.e., in a typical regimen [41]. We took into account the age distribution of parasites at the time of patient presentation (which varies depending on disease severity [32,42,43]), the age-dependence of drug action (CED parameters, from Table 1), the broadening of the age distribution with time (S1 Appendix), and drug-induced growth effects (from Figs 2 and 3). This simulation evaluates the in vivo consequences of the different in vitro drug responses of ring-stage K13 wild-type and mutant parasites (see S1 Appendix). Strikingly, the first and third ART doses given to a hypothetical patient with a PL7-like (resistant) infection result in <10-fold reduction of viable parasites (Fig 7). In contrast, the PL2 (sensitive) strain shows a 50-fold reduction in parasite burden at the corresponding times (Fig 7A, orange curves). As a result, the parasite burden in a PL7-like infection is ~50-fold higher after a three-day treatment. As a consequence of drug-induced synchronization and growth retardation, the parasite age distribution at 72 h consists predominantly of late rings (S7 Fig). Importantly, administration of an additional dose at 72 h is predicted to decrease the parasite load of a PL7-like infection at 96 h to the level observed with a three-day treatment of a PL2-like sensitive infection (Fig 7A, asterisks). In consequence, we predict that a four-day course of ACT will significantly reduce the incidence of treatment failure in areas with ART resistance.

In field studies, Giemsa smears are used to monitor the effectiveness of drug treatment. This approach, however, only detects circulating rings and not mature, sequestered parasites. Our simulation incorporates sequestration and shows that the density of circulating viable parasites will exhibit time-dependent fluctuations over many orders of magnitude, particularly in the period 24–48 h after treatment (Fig 7A, grey curves). These fluctuations are not evident in real patient-averaged data [41], as shown in Fig 7B (symbols), nor in data from individual patients [42]. We posit that this discrepancy arises because our initial simulation assumes that unviable parasites are immediately removed from the circulation. Splenic clearance represents the major in vivo route for removing killed rings [44,45], with unviable parasites persisting for >60 d after ART chemotherapy in splenectomized patients [46]. Our analysis in Fig 3C and 3D shows that unviable rings retain their ring-like morphologies for many hours, indicating splenic clearance would similarly occur over a period of hours. Estimates of the half-life for splenic clearance of dead parasites range from 3 to 6 h [47,48]. Incorporating a clearance half-life of 5 h for removal of unviable parasites (Fig 7B, dashed lines) produces clearance profiles that resemble those measured in patients exhibiting delayed clearance from Pailin (blue symbols), but predicts that both the mutant and wild-type strains would be removed from the circulation at similar rates, despite clear differences in killing (Fig 7A, orange curves). This strongly suggests the existence of additional strain-dependent factors involved in the clearance of unviable parasites.

A possible explanation for a strain-dependent effect is that unviable parasites persist in the circulation (with a ring-like morphology) before changing their physico-mechanical properties sufficiently to initiate splenic clearance. This is consistent with the delayed appearance of pyknotic forms following treatment of cultures with DHA (Fig 3C and 3D), which indicates that unviable parasites retain ring-like morphologies for an extended period. It is also of interest that the half-time to pyknosis is particularly extended in PL7 (K13 mutant) parasites. Indeed, incorporation of an additional strain-dependent term, as well as maintaining a strain-independent splenic clearance rate, permits improved predictions of the observed parasite clearance curves (Fig 7B, solid curves).

This analysis has implications for monitoring parasite clearance times in the field. We anticipate that killed rings that retain a ring-like morphology would persist in circulation and be counted in Giemsa smears. Moreover, unviable resistant parasites would persist for longer in the circulation. Our simulations indicate that in clinical practice, the ring-stage parasites that are detected in Giemsa smears, after ART treatment, likely comprise mainly unviable parasites (Fig 7C, dashed curves). A significant fraction of these will have been rendered unviable after the first dose (dotted lines), especially in fast clearing, sensitive strains. Consequently, the relationship between the in vitro marker of resistance (decreased parasite killing) and the clinical marker of resistance (delayed parasite clearance) is more complex than previously appreciated.

To examine the potential effect on the interpretation of field studies, we simulated a scenario to examine the effect of a split-dose ART treatment on parasite clearance. A recent clinical trial reported that a split-dose ART treatment regimen did not improve parasite clearance times for malaria infections with either ART-sensitive or-resistant *P*. *falciparum* [49]. Indeed, the simulations (see S1 Appendix, S4A Fig) show that splitting the dose will have only a very small effect on the parasite clearance curves. By contrast, there is a very large effect on the number of viable parasites. The simulation predicts that a split-dose regimen should reduce the load of a resistant (PL7-like) infection to a level well below that observed in a sensitive (PL2-like) infection subjected to a standard treatment.

Direct quantitation of circulating viable parasites during drug treatment would complement the current parasite clearance approach for classifying ART resistance in the field. Our simulations indicate that the fraction of circulating parasites that are viable 3 h after commencement of treatment is independent of the typical variation of serum DHA concentrations (*C*_*max*_ = 0.5 to 20 μM [9,49]) and represents a useful parameter for characterizing the in vivo response of a particular strain (S8 Fig). Within this short period of time, there is sufficient difference in loss of parasite viability to distinguish between sensitive and resistant infection, without the complicating effects of significant loss of viable parasites due to sequestration, or from splenic clearance of unviable parasites, which complicates the interpretation at longer times (S8 Fig).

## Discussion

It is generally accepted that ARTs are pro-drugs. That is, they are administered in an inactive form and are activated by reductive cleavage of the endoperoxide ring (see reviews [4–6,50]). The resulting free radicals are thought to react with susceptible groups within a range of parasite proteins and other components, leading to cellular damage and killing. When applied in vitro as clinically relevant short pulses, ARTs are significantly more active against trophozoite-stage parasites than against the mid-ring stage [14]. This likely reflects, in part, the higher availability of iron-containing activators (as a result of hemoglobin degradation) at the trophozoite stage. However, the rate and efficiency of parasite killing will also depend on the ability of the parasite to defend itself against cellular damage. In this work, we have undertaken a careful analysis of the response of K13 wild-type and mutant parasites to ART at different stages and at different exposure times with a view to understanding and overcoming the resistance mechanism.

We found that at low concentrations of DHA, we were able to distinguish cytostatic effects (growth inhibition) from cytotoxic activity (which renders the parasites incapable of reproducing). Interestingly, while different stages and strains of *P*. *falciparum* exhibit very different levels of sensitivity to killing by DHA, the *IC*_*50*_ values for induction of the growth effects are similar. This is consistent with the suggestion that cytostatic effects are initiated as soon as the toxic insult is detected, with downstream killing, if and when the cell defense systems are overwhelmed.

The observed growth retardation is reminiscent of the cellular stress responses reported in other organisms. For example, oxidative and non-oxidative stress events activate an unfolded protein response, leading to shut-down of protein translation and other metabolic pathways [51,52]. While *P*. *falciparum* appears to lack the genes for a classical unfolded protein response [53], it possesses a functional ubiquitin-proteasome system [33,54] and has been shown to undergo eIF2-α–mediated arrest of protein translation, leading to stalling of growth [55].

Consistent with this, we found that the level of ubiquitinated proteins is increased upon exposure to an ART insult, indicating that activated ART damages proteins and initiates a stress response that engages the ubiquitin-proteasome system. By contrast, exposure to a lethal pulse of an anti-folate inhibitor (WR99210) had no effect on the level of ubiquitination. Because ARTs are very short-lived in vivo, the growth stasis that is induced by ART exposure would buy time for the proteasome to degrade ubiquitinated proteins, enabling survival until the ART concentration has declined.

**Fig 8:**
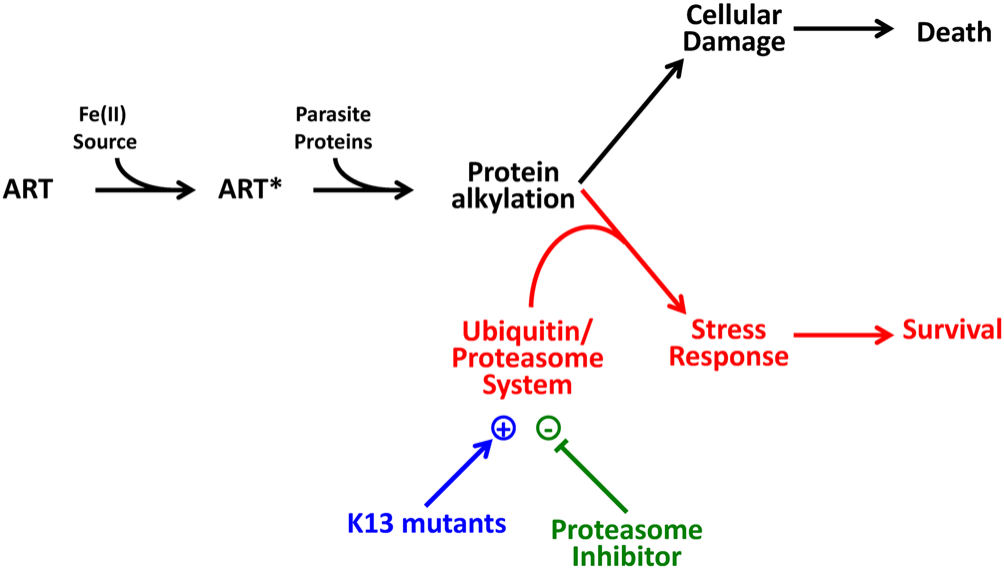
Model of killing and survival-promoting events following treatment with ART. ART is activated by an Fe(II) source (e.g., heme released from hemoglobin degradation or from the cellular labile iron pool) to produce activated ART (ART*), which is reactive, leading to cellular damage and ultimately to parasite death (shown in black). The parasite mounts a stress response, that manifests as growth retardation and engagement of the proteasome-ubiquitin pathway (shown in red). The stress response in K13 mutants is enhanced (shown in blue). In contrast, epoxomicin inhibits the stress response (shown in green), thus promoting parasite death.

Resistance could arise as a result of decreased ART activation or via the mitigation of downstream damage. ART-sensitive and-resistant Pailin strains show similar *K*_*m*_ values for ART-induced killing and similar *IC*_*50*_ values for growth inhibition, indicating similar rates of ART activation. By contrast the extended lag phase before onset of killing and the delayed conversion to the pyknotic state exhibited by K13 mutants, as well as the lower levels of ubiquitinated proteins and the synergism with proteasome inhibitors, are consistent with the suggestion that an enhanced cellular stress response underlies resistance. We present a possible model for ART action and the cell stress response in Fig 8, in which death occurs when the level of damage overwhelms the parasite's proteasome system.

As predicted by this model, we found that proteasome inhibitors, such as epoxomicin, Carlifzomib, and Bortezomib, markedly synergize the action of DHA. In the laboratory strain 3D7 this effect is particularly marked in the mid-ring stage of development, when the parasite shows low sensitivity to DHA, but is not evident in the very early ring stage, when the parasite exhibits ART hypersensitivity. In the K13 mutant strains, PL1, 5, and 7, the synergism is particularly marked at the very early ring stage when these parasites are especially resistant to DHA. In an effort to distinguish the role of the *K13* gene product from other contributing genetic differences, we examined the level of synergism in a Cambodian K13 mutant (Cam 3.II) and a genetically matched K13 wild-type (reverted) transfectant. The revertant shows very early ring stage sensitivity that is similar to that that observed for the Pailin wild-type strain (PL2). Epoxomicin markedly synergized the activity of DHA against the very early ring stage of the K13 mutant, but also increased its activity against the revertant. This suggests that a proteasome-engaging cell stress response is involved in protecting both sensitive and resistant parasites from the action of ARTs, but that this response is more effective in the K13 mutant parasites. Our data suggest that proteasome inhibitors could provide a synergistic combination with ARTs that would enhance their effectiveness against both K13-mutant and wild-type parasites.

K13 shares some sequence similarity with KEAP1 and KLHL8, which are involved in E3 ubiquitin ligase complexes that regulate the cytoprotective and developmental responses in mammalian systems [56]. A recent transcriptomic analysis showed up-regulation of the unfolded protein response in K13 mutant parasites, including genes involved in protein folding, unfolded protein binding, protein export, post-translational translocation, signal recognition particle, endoplasmic reticulum retention sequences, the proteasome, and the phagosome [32]. Taken together with our data, this strongly indicates a role for enhanced proteostasis mechanisms as the basis for ART resistance in *P*. *falciparum*.

The transcriptomic analysis of Mok et al. also suggested that, at a population level, resistant parasites exhibit decelerated development at the ring stage. We found that parasites (e.g. PL1 and PL2) that exhibit similar lifecycle profiles (as judged by Giemsa analysis) can have very different responses to ARTs. This indicates that while decelerated development may be a contributor, other factors are also important. Further work is required to fully elucidate the role of K13 in enhancing the cell stress response.

Importantly, our work suggests that a proteasome inhibitor could be used to synergize the activity of ARTs in vivo, and potentially to overcome resistance. Carfilzomib and Bortezomib are FDA-approved for the treatment of multiple myeloma [57] and inhibitors that specifically target the plasmodial proteasome have been identified [39,58,59]. These compounds show low toxicity in human cell lines [59] and limited toxicity in mice [40,58]. In agreement with previous reports, we find that Carfilzomib shows only weak antimalarial activity, when used alone, against the ring-stage of a mouse model of malaria [40]. In contrast we observed marked synergism of the action of DHA by Carfilzomib, against *P*. *berghei* in vivo, particularly in reducing the parasite burden in reticulocytes, the preferred host blood cell. While further work is needed to determine the efficacy of proteasome inhibitors as ART-synergizing-agents in patients, this work offers a potential avenue to overcome ART resistance.

We analyzed the concentration and exposure-time—dependence of the response of K13 mutant and wild-type parasites in terms of the CED model, enabling for the first time prediction of in vivo parasite clearance profiles from in vitro assessments of ART sensitivity. Our modelling indicates that slower in vivo clearance of resistant strains may reflect both decreased killing of parasites and a slower rate of clearance of parasites that have been rendered unviable. Prolonged circulation of unviable (but morphologically unchanged) ring-stage parasites will complicate the analysis of Giemsa-stained peripheral blood smears. This should be considered in the analysis of parasite clearance curves in surveillance studies. While current methods for monitoring clearance curves are adequate for detecting reduced sensitivity of infecting parasites, they largely reflect parasite killing during the first treatment dose and may not be suitable for evaluating the effectiveness of new antimalarials or alternative treatment regimens. For example, our simulations predicted that splitting the ART dose would substantively decrease the level of viable parasites but would have no effect on parasite clearance curves. This suggests that a recent clinical study of a split-dose regimen [49] may have underestimated its effectiveness.

Methods enabling direct monitoring of parasite viability after ART treatment are needed. Our simulations suggest that a suitable parameter to measure is the fraction of circulating viable parasites at a time point 3 h after the commencement of treatment. Our data suggest that culturing drug-exposed ring-stage parasites in a drug-free environment for 35 h will enable the ready distinction of the viable (trophozoites) from non-viable (rings and pyknotic) parasites and readily distinguish K13 wild-type from mutant parasites. We anticipate that this approach can be made field-adaptable by culturing washed blood collected from patients 3 h after treatment. Assessment of parasites with a trophozoite-like morphology after 30–40 h in culture would provide a robust and direct measure of the likelihood of treatment failure.

Another important implication of our findings is that extended ACT treatment courses could reduce the viable parasite load to curative levels in ART-resistant falciparum malaria, since further parasite maturation on the fourth day of treatment will render them much more sensitive to ART. This is consistent with recent trial data [13] showing 97.7% efficacy of a six-day treatment course, and adds further support to calls for urgent testing of extended treatments in affected areas. The implementation of new treatment regimens could help battle ART resistance. This is critical, given that a recent modelling study suggest that even a 30% ACT failure rate worldwide would result in more than 116,000 additional deaths per year, and US$385 million of annual productivity losses [60].

## Materials and Methods

### Isolation and Culture Adaptation of Cambodian *P*. *falciparum* Strains

*P*. *falciparum* isolates were collected from adult patients enrolled in clinical trials conducted between 2009 and 2010 in Pailin Referral Hospital in western Cambodia as described previously [21]. Strains were adapted to cultivation in vitro in RPMI supplemented with glutamine and 10% human serum. K13 propeller genotyping of strains were performed as previously described [21]. Parasite lines were expanded and aliquots frozen. All analyses were performed within 4 wk of thawing. All strains are independent isolates based on typing of MSP1, MSP2, and GLURP, and all exhibit a PfCRT mutant genotype (CVMNT), as typical for this region [21,61]. A slow-clearance isolate from Pursat province in Cambodia (RF967/ Cam3.II), harboring the R539T mutation, and a cloned reverted line (Cam3.IIrev), carrying the wild-type allele, were generated as described elsewhere [20]. In vitro culturing, including generation of very tightly synchronized cultures, was carried out as previously described [14,28]. All data presented pertains to cultures synchronized to a 1-h window. The parasite ages defined correspond to the average post-invasion (p.i.) age at the beginning of each drug pulse. Parasite age distributions during the schizont-to-ring transition were quantitated from Giemsa smears by periodically examining the number of schizonts and rings of untreated cultures during the transition, as previously described [14].

### Preparation of DNA and Genome Sequencing

Genomic DNA was extracted using the ISOLATE II Genomic DNA kit (Bioline, United Kingdom). Genomic DNA (200 ng) was sheared using an ultrasonicator, and Illumina TruSeq Nano DNA library preparation was carried out according to the Manufacturer’s instructions. The libraries were pooled and run with paired-end 300 bp reads on a MiSeq platform over 600 cycles of sequencing using the MiSeq Reagent kit v3 (Illumina, United States). All raw reads have been submitted to the European Nucleotide Archive (ENA) under the accession number PRJEB8074. Following adapter trimming, the raw reads were mapped to the reference 3D7 genome (version 3.0) using BWA mem and filtered for mapping quality score of at least 30. Duplicate reads were marked and removed using SAMtools and Picard, and the reads were realigned around INDELS using Genome Analysis Tool Kit (GATK) RealignerTargetCreator (Broad Institute, US). Following recalibration of base quality scores, SNPs were called using GATK UnifiedGenotyper, and hard-filtering of SNPs was performed to obtain high-quality variants. SNPs that failed any of the following cut-off filters were removed from the analysis: depth of read <5, variant quality as function of depth QD <2.0, strand bias (*P*) <10^-6^, mapping quality <40, MappingQualityRankSum <−12.5, ReadPosRankSum <−8.0. Genetic variants were annotated using snpEFF.

### Assessment of Parasite Viability and Drug-Induced Growth Effects by Flow Cytometry

Drug pulse assays used to estimate *LD*_*50*_ and *V*_*min*_ have been described previously [14,28]. Parasite viability following a drug pulse is defined as the fraction of the parasite population that survives drug exposure and is able to enter the next parasite cycle. Viability was determined by measuring the parasitemia in the parasite cycle following the drug pulse. For this, parasites were fluorescently labelled with the RNA-binding dye SYTO-61 and the parasitemia quantitated by flow cytometry [62]. Viability was calculated in relation to parasitemia in the "untreated parasite" control (parasites not exposed to drug) and "kill" control cultures. The latter refer to parasites maintained under constant drug pressure (>100 times the *LD*_*50*_ *(48 h)*) for 48–96 h to ensure quantitative killing of parasites. The sensitivity limit for viability using this assay is 5%. *LD*_*50*_ is the drug concentration producing 50% viability. *V*_*min*_ is defined as the viability at saturating drug concentration and was established by examining viability at the highest drug concentration employed in a particular assay.

SYTO-61 labelling of parasites was also used to measure growth effects related to drug treatment. Such measurements require labelling to be performed when the untreated parasite control culture has progressed to mid-–late trophozoite stage in the cycle following the drug pulse. This ensures that the SYTO-61 signal from viable parasites exhibiting growth effects show a measurable decrease compared to the no drug control. The SYTO-61 frequency histogram at a particular drug concentration was corrected for the presence of unviable/dead parasites by subtracting the appropriate amount of the "kill" control histogram based on the measured viability at that drug concentration. Corrected SYTO-61 signals refer to the median value of the viable parasite population and were calculated from the corrected histograms, and are only reported for sample measurements exhibiting the following criteria: parasitemia >0.3%, number of parasites measured >500, and fraction of total parasites measured that are viable >0.7.

### Ubiquitinated Protein Analysis

Trophozoite-infected RBCs (25–35 h) or uninfected RBC (3% hematocrit) were incubated with 6 μM QHS or 20 nM WR99210 for 90 min at 37°C. Cells were pelleted, washed three times in PBS supplemented with anti-protease mixture (APM), 20 mM *N*-ethylmaleimide (NEM), 2 mM PMSF, 0.5 mM EDTA, and complete mini EDTA-free protease inhibitor mixture (Roche). Cell pellets were resuspended in 10 volumes of 0.15% (w/v) saponin in PBS for 10 min on ice. Cells were pelleted and washed twice with PBS + APM. Parasite pellets were subjected to SDS-PAGE (4%–12% acrylamide; Life Technologies), transferred to nitrocellulose (iBlot, Life Technologies), and probed with polyclonal rabbit anti-ubiquitin IgG (Z 0458, Dako, 1:100 dilution in PBS), followed by goat anti-rabbit IgG coupled to horseradish peroxidase. Chemiluminescence was detected using a LAS-3000 Imaging System. Membranes were stripped with 0.2 M glycine, 0.1% (w/v) SDS, 0.01% (v/v) Tween-20, pH 2.2, and re-probed with rabbit anti-*Pf*GAPDH [63]. Initial control experiments compared the signal in uninfected RBCs and very early ring-stage—infected (1–2 h p.i.) RBCs (5% parasitemia). Densitometric analysis of the region of the gel between 80 and 200 kDa was performed using ImageJ software. The data were corrected for background and for loading based on the *Pf*GAPDH signal.

### Mouse Studies

Mice were housed with strict temperature control (21°C) and under a 12:12 light:dark cycle. All mouse experiments were approved by the Animal Ethics Committee at Macquarie University (ARA 2012/018) and conformed to the National Health and Medical Research Council guidelines. For rodent malaria infection, 250 μl of thawed *P*. *berghei* ANKA parasitized blood were intraperitoneally injected onto SJL donor mice. Once the donor mice reached 5%–15% parasitemia, the mice were exsanguinated and the blood was diluted in Kreb’s buffered saline [64] at a dose 1x10^6^ parasitized RBCs. The parasitized RBCs were injected into the peritoneal cavity of BALB/c female mice. After inoculation, the mice were monitored daily or twice daily using tail bleed smearing or flow cytometry using JC-1 dye gated on TER119, cd71, and cd45 [65]. For the drug administration, infected BALB/c mice (~21 g) were treated with intravenous doses of Carfilzomib (0.5, 1, and 1.5 mg/kg), intra-peritoneal doses of DHA (5, 10, 15, and 20 mg/kg), a combination of Carfilzomib and DHA (5 or 10 mg/kg of Carfilzomib, with respectively 5 and 10 mg/kg of DHA), or vehicle (10 mM citrate buffer, 20% Kleptose for Carfilzomib and 60% dimethyl sulfoxide, 40% Polysorbate 80 for DHA). The mice were drug-injected for three consecutive days, starting day 3 post-inoculation. A Student’s one tailed *t* test was performed and corrected with a Bonferroni procedure for multiple testing to investigate the statistical differences between drug treatments.

### Kinetic Data Analysis and Simulations

Parasite viability (*V*) as a function of drug concentration (*C*) and drug exposure time (*t* ^*e*^) were analyzed according to the CED model [14]:

$$V\left(C,t^{e}\right)={\left[1+{\left(\frac{Ct^{e}}{t_{50}^{e,sat}\left(K_{m}+C\right)}\right)}^{\gamma }\right]}^{-1}$$

where *K*_*m*_ is the drug concentration resulting in half the maximum effective dose, *γ* is the slope of the sigmoidal function, and $t_{50}^{e,sat}$ is the minimum time required to kill 50% of the parasites (at saturating drug concentrations). The model was fitted to the data, using Microsoft Excel with the Solver add-in. The method employed for simulating parasite load during a three-day course of ART monotherapy is presented in S1 Appendix. An Excel spreadsheet for the simulation of parasite clearance is also supplied (S1 Spreadsheet).

## Supporting Information

S1 Appendix Simulation of in vivo parasitemia during DHA monotherapy and split-dose therapy. (PDF)

S1 Data Numerical values for all figures. (XLSX)

S1 Fig Effect of drug exposure time on dose response profiles of PL2 (wild-type) and PL7 (R539T) strains. Measurements were carried out as described in Fig 1C, by exposing parasites of defined ages to DHA for the time periods indicated in the legends. Viability is defined as the fraction of the parasite population that survives drug exposure and is able to enter the next parasite cycle. The parasites were early rings (2 h p.i., top panel), rings (7 h p.i., middle), or trophozoites (34 h p.i., bottom). Solid curves represent the best fits with the CED model parameters shown in Table 1. Reliable parameters could not be obtained with the set of measurements represented by the dashed line because the drug dose was already saturating at the shortest exposure time (30 min). Error bars correspond to the range of duplicates.
(TIFF)

S2 Fig Interaction of epoxomicin with DHA in PL2, PL5, and PL7 strains at different stages. PL2 (A), PL5 (B), and PL7 (C) parasites at different stages (indicated by their p.i. age) were subjected to 3-h pulses in the presence of different combinations of DHA and epoxomicin (Epo). The left side of each panel shows the influence of sublethal concentrations of epoxomicin (indicated in figure) on the dose-response profile of DHA (blue symbols). The right side of each panel presents the isobolograms for the epoxomicin-DHA pair at the 50% *LD*_*50*_ level. The dashed line is plotted between the *LD*_*50*_ of each drug used alone, emphasizing the concave nature of many of the isobolograms. The absence of a line indicates the *LD*_*50*_ value of one of the drugs was outside the range of concentrations examined. Error bars, where present, correspond to the range of duplicates.
(TIFF)

S3 Fig Interaction of epoxomicin with DHA in the matched K13 mutant and wild-type transfectants strains. Cam3.II (R539T) (A) and a reverted transfectant in which the *K13* wild-type genotype has been restored (Cam3.II_rev) (B) were synchronized to a 1-h window and subjected to 3-h pulses in the presence of different combinations of DHA and epoxomicin (Epo) at the following times p.i.: 1 h (very early ring, vER), 7 h (ring, R), and 27 h (troph, T). The left panels show the dose-responses to DHA and the influence of sub-lethal concentrations (indicated) of epoxomicin. The right panels show the isobolograms for the epoxomicin-DHA pairs at the 50% lethal dose level. Error bars, where present, correspond to the range of duplicates.
(TIFF)

S4 Fig Interaction of carfilzomib with DHA in PL2, PL1, PL7, and PL5 strains at different stages. PL2 (A), PL1 (B), PL7 (C), and PL5 (D) parasites at different stages (indicated by their p.i. age) were subjected to 3-h pulses in the presence of different combinations of DHA and carfilzomib (Carf). The left side of each panel shows the influence of sublethal concentrations of carfilzomib (indicated in figure) on the dose-response profile of DHA (blue symbols). The right side of each panel presents the isobolograms for the carfilzomib-DHA pair at the 50% *LD*_*50*_ *(3h)* level. The dashed line is plotted between the *LD*_*50*_ *(3h)* of each drug used alone, emphasizing the concave nature of many of the isobolograms. The absence of a line indicates the *LD*_*50*_ *(3h)* value of one of the drugs was outside the range of concentrations examined. Error bars, where present, correspond to the range of duplicates from an experiment performed in singlicate.
(TIFF)

S5 Fig Interaction of Bortezomib with DHA in the PL7 strain at the trophozoite stage. PL7 parasites (3-h window, mid-trophozoites) were subjected to 3-h pulses in the presence of different combinations of DHA and Bortezomib (Bort). The left panel shows the dose-responses to DHA (diamonds) and the influence of sub-lethal concentrations (indicated) of Bortezomib. The right panel shows the isobologram for the Bortezomib-DHA pair at the 50% lethal dose level. Error bars, where present, correspond to the range of duplicates.
(TIFF)

S6 Fig In vivo synergy between DHA and Carfilzomib against infected reticulocytes. Balb/c mice were infected with 10^6^ parasites (*P*. *berghei*) and treatment was initiated at ~1% parasitaemia (i.e., day 3), indicated with an arrow. (A) Delivery of vehicle alone is associated with high parasitemia (in RBCs), while DHA alone has a significant effect on parasite burden at all doses. (B) The same data displayed on an expanded scale reveals that drug treatment with DHA at doses 5 and 10 mg/kg has a moderate effect on parasite growth, whereas the doses 15 and 20 mg/kg totally abrogate parasite growth. (C) DHA (5 mg/kg) + Carfilzomib (0.5 mg/kg) modestly prevents parasite growth in reticulocytes, whereas DHA (5 mg/kg) + Carfilzomib (1 mg/kg) drastically reduces the parasite burden after treatment. (C) DHA (10 mg/kg) + Carfilzomib (0.5 or 1 mg/kg) abrogate the growth of *P*. *berghei* parasites in reticulocytes. Error bars represent SEM, and * and *** represent, respectively, *p* < 0.05 and *p* < 0.01 after Bonferroni correction for multiple testing.
(TIFF)

S7 Fig Age distribution (simulated from model) of viable parasites during the course of drug treatment (0, 24, 48, and 72 h post initial dose) for the PL2 (red) and PL7 (blue) infections presented in Fig 7. (TIFF)

S8 Fig Measurement of the circulating viable fraction during ART therapy can distinguish between sensitive and resistant strains. Simulations for PL2 (red) and PL7-like (blue) infections were performed as described in Fig 7 but using various values for *C*_*max*_ (0.5, 2, and 20 μM corresponding to dotted, dashed, and solid lines, respectively). The circulating viable fraction at each time is the total number of circulating viable parasites divided by the total number of circulating parasites (i.e., including unviable parasites). Arrows indicate that this parameter at 3 h is insensitive to the value of *C*_*max*_ and can distinguish between the two strains.
(TIFF)

S1 Spreadsheet Excel Spreadsheet for simulation of parasite clearance curves. The Parasite Clearance Simulation can be downloaded as a separate file.
(XLSX)

S1 Table Parameters relating to the quality of the Illumina MiSeq genome sequence data. (PDF)

S2 Table SNP analysis of Pailin strains at loci reported to be associated with differences in ART sensitivity. Kelch 13 (K13), *P*. *falciparum* Multi-Drug Resistance Gene-2 (MDR2), apicoplast ribosomal protein (ARPS10), ferredoxin (FD), PfCRT, protein phosphatase (PPH), phosphoinositide-binding protein (PIB7), Multi-Drug Resistance Gene-1 (MDR1), Ubiquitin Binding Protein-1 (UBP1), Multidrug Resistance Protein-1 (MRP1), cyclic nucleotide-binding protein (cNBP) and the 3′ UTR of the DNA polymerase catalytic subunit. No artemisinin resistance associated mutations were observed in RAD5 or SERCA.
(PDF)

S3 Table Parameters used in simulation presented in Fig 7A. (PDF)

## Abbreviations

ARTs: artemisinins
ACTs: ART combination therapies
DHA: dihydroartemisinin
*LD_50_*: 50% lethal dose
p.i.: post-invasion
QHS: qinghaosu
RBC: red blood cell
*V_min_*: minimum viability
