# Supplementary material for: Targeting the Cell Stress Response of Plasmodium falciparum to Overcome Artemisinin Resistance
Source: PLoS Biol. 2015 Apr 22;13(4):e1002132. doi: 10.1371/journal.pbio.1002132 (PMC4406523; doi:10.1371/journal.pbio.1002132)
Supplement: S1 Appendix — (PDF) [file pbio.1002132.s001.pdf]

Appendix S1. Simulation of *in vivo* parasitemia during DHA monotherapy and split dose therapy

Derivation of Cumulative Effective Dose (CED) model parameters throughout the intraerythrocytic cycle

The CED model is described by the Equation below [1]

$$V(C,t^e)=\left[1+\left(\frac{Ct^e}{t_{50}^{e,sat}\left(K_m+C\right)}\right)^{\gamma}\right]^{-1}$$

where V is viability, C is the drug concentration, K_m is the drug concentration resulting in half the maximum effective dose, t^e is the drug exposure time, γ is the slope of the function and $t_{50}^{e,sat}$ is the minimum time required to kill 50% of the parasites (at saturating drug concentrations).

The parameters derived from analyzing the *in vitro* concentration and time dependence of DHA action according to the CED model ($t_{50}^{e,sat}$, γ, and K_m) can be used to determine parasite viability during the course of a drug pulse that mimics the *in vivo* pharmacokinetics of DHA [1]. In order to take into account the presence of different parasite ages *in vivo* during treatment, it is necessary to account for variations in the parasite's response to DHA at different stages of the blood cycle. In the present study, we determined these parameters from *in vitro* viability measurements of parasites exposed to drug for a range of time periods between 0.5 h and 9 h. These detailed studies were performed at three parasite stages: early rings (2 h p.i.), late rings (18 h p.i.) and trophozoites (34 h p.i.) (Figure 1C, Figure S1 and Table 1). These studies allowed accurate determination of the CED model parameters at these particular ages.

To estimate CED model parameters for ring-stage parasites of other ages we took a two-stage approach. First, we used information from the dose-response profiles measured with a fixed drug-exposure time of 3 h but at high frequency across the ring stage (Figure 1A,B of the main manuscript shows $LD_{50}(3h)$ and $V_{min}(3h)$ values derived from this data) to assess the *shape* of the age dependency for each of the CED parameters. In order to estimate the CED parameter values using the data from a single 3 h drug pulse, the parameter space searched in the nonlinear regression was constrained to within 10% of the largest and smallest parameter values derived from the aforementioned detailed studies at 2 h and 18 h p.i.. This provided us with sufficiently well determined estimates of the CED model parameters to conclude that $t_{50}^{e,sat}$, K_m and γ were well described by exponential, Lorentzian and linear functions, respectively (Figure SA1). Secondly, these functional forms of the CED model parameters versus age relationships, combined with robust indicators of CED parameter variation, such

as the peak in K_m at an approximate parasite age of 16-20 h p.i., were used to interpolate between the two accurate age-specific measurements (ages 2 h and 18 h p.i.) to derive the final age-dependent formulas for CED model parameters for ring-stage parasites (Figure SA2). For ages less than the earliest measurement (2 h p.i) we assumed that the CED parameters remained constant (Figure SA2)

For trophozoites and schizonts, we took a simpler approach, assuming that CED model parameters were constant throughout most of the mature stages (see Figure 1A,B and [1]) and then "decayed" with a half-life of 1.5 h to the values observed in the earliest rings. This accounted for the increasing $LD_{50}(3h)$ and $V_{min}(3h)$ values of late schizonts for the resistant strains (see *b* panels in Figure 1A,B and associated text in main manuscript). It was not possible to extract reliable model parameters for the response of PL2 strain trophozoites to DHA because the rapid drug response (see Figure S1) resulted in a high degree of correlation between the parameters precluding their direct determination. The model parameters for the PL7 strain were therefore used for the trophozoite stage of both strains. As a consequence the simulations of PL2- and PL7-like infections, derived from these CED model parameters, are confined to examining differences due to the different ring-stage responses of the different strains. This is valid because it is clear, even in sensitive strains, that it is the ring-stage response that is "limiting" with respect to the *in vivo* ART drug response, and thus decreased ring-stage sensitivity is responsible for the delayed clearance times (this study, and [1]).

The measured *in vitro* cycle times for strains PL1, 2, 5 and 7 were 49, 52, 59 and 57 h, respectively, and the ring to trophozoite transition occurred at 27, 27, 29 and 29 h p.i. respectively (Figure 1A,B). For the *in vivo* simulation, the cycle time was constrained to 48 h to mimic *in vivo* cycle times [2,3] and the ring to trophozoite transition assumed to occur at 26 h p.i for each strain. The calculated CED model parameters across the intraerythrocytic cycle for this scenario are shown in Figure SA2.

The LD_{50} and V_{min} for a particular *in vitro* exposure time (t^e) (at constant drug concentration) can be calculated from the model parameters [1]:

$$V_{\min}(t^e) = \left[1 + \left(\frac{t^e}{t_{50}^{e,sat}}\right)^{\gamma}\right]^{-1} \qquad \text{[eq. SA1]}$$

$$LD_{50}(t^e) = \frac{t_{50}^{e,sat} K_m}{t^e - t_{50}^{e,sat}} \qquad \text{[eq. SA2]}$$

and are shown in Figure SA3 for a 3 h drug pulse. The predicted behavior of the strains across the intraerythrocytic cycle, derived from the CED model parameters, follows the trends observed in experimental analysis of a 3 h drug pulse, as shown in Figure 1A.

Modelling viability during DHA monotherapy

The DHA pharmacokinetic profile was simulated using the median population value determined in a 2009 Pailin study [4], in which a dose of 2 mg/kg was administered. The parameters used were a linear rise time of 1 h to a C_{max} value of 2820 nM, and a subsequent elimination half-life of 51 min. Patients with uncomplicated falciparum malaria presenting for treatment are generally assumed to possess ring-stage infections [2,5] though it is not clear to what extent such infections are synchronous. The initial age distribution of the parasites was thus modelled as a Gaussian distribution with an average parasite age, μ_{age}, and standard deviation, σ_{age}. Parasites with negative ages in these distributions correspond to schizonts that have yet to enter the next cycle and hence had 48 h added to their ages and their numbers divided by the replication factor (assumed to be 10 in the simulation). We used the μ_{age} and σ_{age} values of 7 and 7 h which are typical of the values reported in an intra-host modelling study from the Pailin and Wang Pha regions [2]. Altering these values to represent various types of ring-stage infections does not affect the conclusions presented in the main manuscript.

For each drug administration, the viability of each parasite subpopulation (*i.e.* of a specific age with a resolution of 1 h) as a function of time was calculated numerically, using the $t_{50}^{e,sat}$, γ, and K_m parameters associated with that parasite subpopulation [1]. The overall viability is thus determined by the distribution of subpopulation ages at the start of treatment, which concurs with the methodology used to measure and analyze parasite viabilities in the current study. The number of viable parasites in each subpopulation at each time point is calculated by multiplying the viability at each time point by the number of the parasites present at the start of treatment. Viable parasites that enter the next cycle through asexual reproduction have their numbers multiplied by the replication factor. The age-distribution of viable parasites present 24 h after a particular treatment is used as the input for the next treatment.

Giemsa smears from patients only reveal circulating parasites (usually young rings) and not the mature forms, which are sequestered. The number of *viable* circulating parasites of a particular age was calculated based on the parasite age (a), the age at which sequestration begins (a_b) and the age at which 50% of parasites are sequestered (a_{50}) according to $2^{(a_b-a)/(a_{50}-a_b)}$ (for $a > a_b$), with all parasites with ages $< a_b$ assumed to be circulating [2], and using values of 15 and 18 for a_b and a_{50}, respectively.

This basic form of the simulation model was extended to take into account two other observations from this study: growth retardation and the broadening of the age distribution during *in vitro* culturing.

The majority of the parasites that survive a drug pulse are early ring-stage parasites, and we demonstrated that viable parasites surviving a drug pulse exhibit growth retardation with respect to untreated parasites. For early ring-stage parasites, we measured the extent of retardation to be 6 h (see Figure 3A,B and associated text in main manuscript). Without loss of generality (*i.e.* for numerical convenience only) growth retardation was assumed to occur linearly over a period of twice the degree of the growth retardation (i.e. over 12 h for a 6 h retardation).

Continuous *in vitro* culturing of parasite cultures results in broadening of the age distribution over time. We measured the degree of broadening in this study (*e.g.* the schizont to ring transition shown in the *b* panels in Figure 1). In agreement with a previous study [1], ~ 80% of parasites that are synchronized to a 1-h window undergo the schizont to ring transition over a period of ~ 4 h in the next cycle (*i.e.* 48 h later). Application of a drug pulse leads to additional broadening. For the simulation, broadening of the age distribution was calculated every 24 h (*i.e.* prior to the administration of the subsequent drug treatment) and the broadened age distribution was used as the input for the next round of treatment. Broadening was achieved by redistributing the number of parasites in each 1-h age window to other age windows using a normalized Gaussian distribution with a standard deviation σ_{broad}. For parasites redistributed into negative age windows, this was performed as described above for the initial distribution of parasite ages at time zero. Parasites placed into age windows ≥ 48 h correspond to schizonts that form rings in the next cycle. In these cases, the number of these parasites was multiplied by the replication factor and 48 h was subtracted from their age.

The CED model can be used to determine when a parasite population becomes unviable (*i.e.* when it receives a sufficient cumulative effective dose so that it can no longer enter the next parasite cycle), but provides no information as to when such circulating parasites would disappear from the circulation. In order to account for the persistence *vs* clearance of unviable parasites *in vivo*, we incorporated clearance components into the model for the simulation. This was achieved by calculating the number of unviable parasites generated during each 1-h window of the treatment regime. The unviable parasites were assumed to remain sequestered, in the same way as viable parasites (if that was their status at the time they were generated), but to show no further ageing. The unviable parasites were cleared according to either a simple first order decay representing splenic clearance (rate constant k_{spl}) or according to a 2-step process. For the latter case, the first step was defined by the first order rate constant k_{str} which is strain-dependent and represents the physio-mechanical changes required for the unviable parasite to be amenable to splenic clearance (see main text). The second step was defined by the first order rate constant k_{spl} which is independent of the strain and models the process of splenic

clearance (see main text). The number of circulating unviable parasites generated at time T after treatment (U_{0T}^{circ}) will thus decay according to:

$$U_T^{circ}(t) = U_{0T}^{circ}\left(k_{str}e^{-k_{spl}(t-T)} - k_{spl}e^{-k_{str}(t-T)}\right)\Big/\left(k_{str} - k_{spl}\right) \text{ for } t \geq T \qquad \text{[eq. SA3]}$$

where U_T^{circ} is the number of the circulating unviable parasite population generated at time T that are present at time *t* following the start of treatment.

The parameters used for the simulations presented in Figure 7 are summarized in Table S3.

An interface that can be used to vary the parameters for the simulation of parasite load during a 3-day course of ART monotherapy using an Excel spreadsheet is available as Supplementary Material (Spreadsheet S1). The user can choose the relevant strain for the simulation (PL2 or PL7 for K13 wildtype or mutant, respectively) and vary (i) the DHA pharmacokinetic parameters in the patient, (ii) the parasite age distribution at the time of presentation, (iii) the parasite replication rate, (iv) the timing of sequestration, (v) the strain-dependent rate of changes upon drug treatment leading to splenic recognition, (vi) the strain-independent rate of splenic clearance of unviable parasites, (vii) the rate of broadening of parasite age distribution with and without treatment, and (viii) the degree of growth retardation during drug treatment.

Simulation of in vivo parasitemia during split dose therapy with DHA

We used a similar approach to compare parasite loads during a standard 3-day ART treatment (2 mg/kg artesunateevery 24 h) with those during a split dose 3-day ART treatment (1 mg/kg artesunate every 12 h) for infections with PL2 and PL7 strains. The parameter chosen are based on those from a clinical trial [6]. The general approach was similar to that described above except (i) the C_{max} value for the split-dose simulation was half the value (1410 nM) of the standard simulation (2820 nM) and (i) the broadening of the parasite age distribution for both the standard and split-dose treatment regimens were calculated every 12 hours using a σ_{broad} value of 2 h (compared to a value of 3 h every 24 h in the previous simulations). The half-lives for the clearance of unviable parasites parasite were the same as those used in Figure 7C. An excel spreadsheet pertaining to the split-dose simulation is available on request.

These simulations show that splitting the dose has only a very small effect on the parasite clearance times (Figure SA4, dashed vs solid black curves), a result that is similar to that observed in the clinical trial [6]. Importantly, the simulation shows that the small changes in parasite clearance times with the different treatment regimens do not reflect the underlying large differences in viable parasite loads (dashed vs solid orange curves). In particular the simulation predicts that a split dose regimen of a PL7 infection should reduce parasite loads to levels far below those observed in a PL2

infection subjected to a standard treatment regimen. Interestingly, the PL7 simulation indicates little difference between viable parasite loads following standard and split-dose treatment regimens after 36 h (compared dashed and solid orange curves). This is because of drug-induced growth retardation which means the resistant rings are being repeatedly hit by the drug during the first three of the 12 h treatments. It indicates that the overall success of the split-dose treatment regime arises because it ensures that the drug sensitive trophozoites are hit three times by the drug leading to a substantial decrease in the viable parasite load.

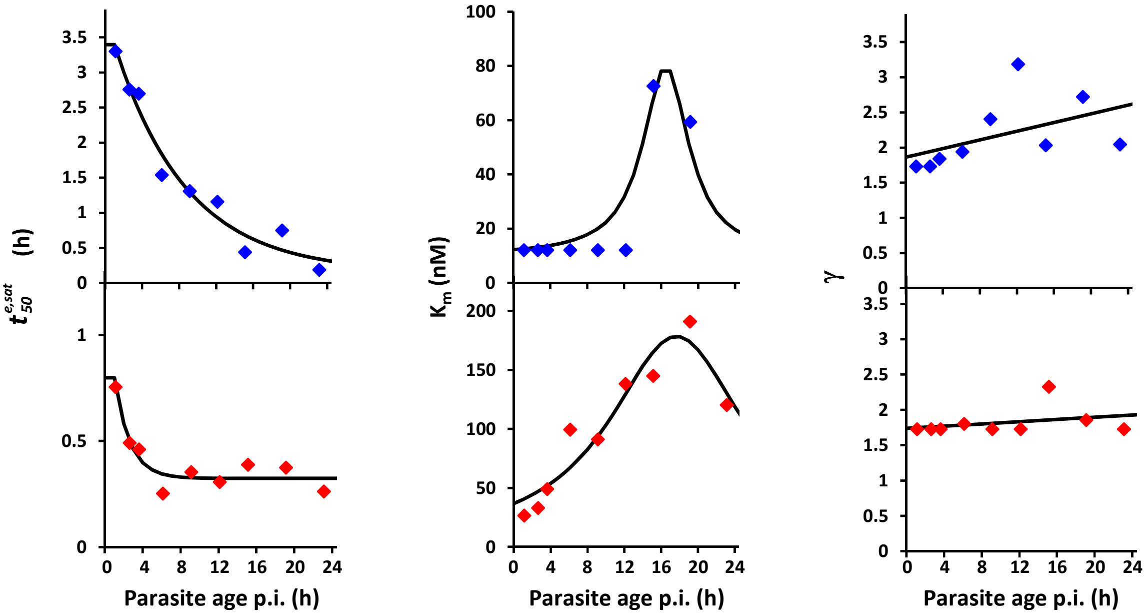

Figure SA1. Variation of CED model parameters with parasite age during the ring-stage. Dose-response profiles across the ring-stage were measured for 3 h DHA pulses ($LD_{50}(3h)$ and $V_{min}(3h)$ values derived from this data are presented in Figure 1A of the main manuscript). CED model parameters from each dose-response profile (symbols) were estimated by fitting the CED model to each dose-response profile as described in the text. Shown are the parameters obtained for the PL2 (red symbols) and PL7 (blue symbols) strains. The curves represent empirical fits to the data and illustrate the way these parameters change with parasite age. The forms of the curves indicate exponential ($t_{50}^{e,sat}$), Lorentzian (K_m) and linear (γ) dependencies.

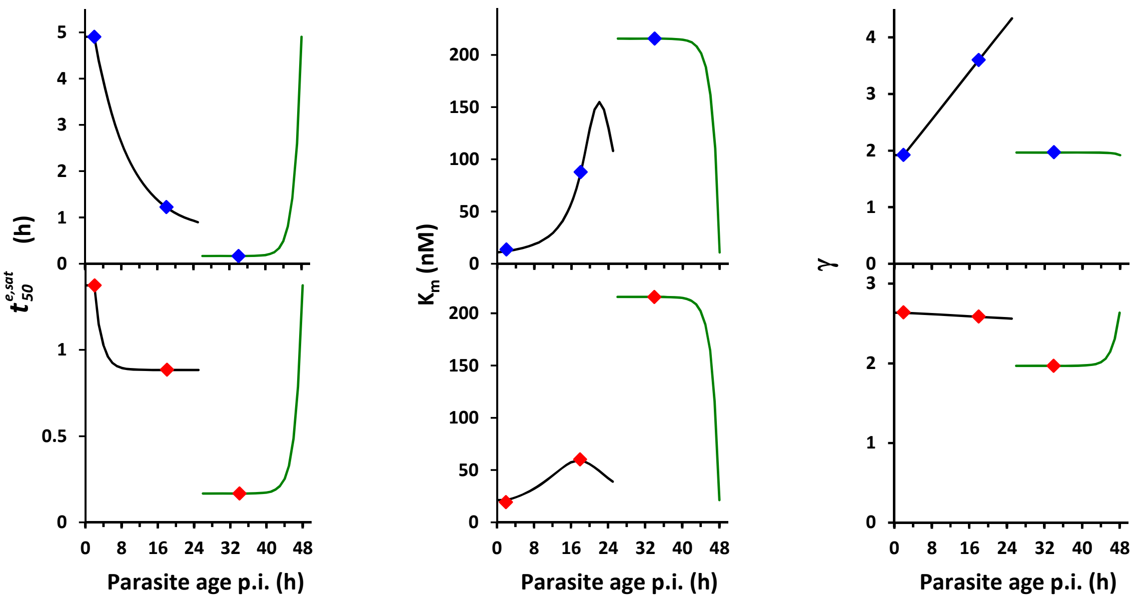

Figure SA2. CED model parameters at different stages of the intraerythrocytic cycle. Model parameters for PL2 (red symbols) and PL7 (blue symbols) were derived by fitting the CED model to dose response profiles of early ring, late rings and trophozoites (2, 18 and 34 h p.i, respectively) measured at different exposure times. The predicted values of the model parameters at other ages at ring stages (black curves) and trophozoite stages (green curves) were derived as described in the text. The blood stage lifecycle was constrained to 48 h [3] and the ring to trophozoite transition occurs at 26 h p.i producing a discontinuity at this time point. This transition would be smoother *in vivo* reflecting the behavior of populations of parasite with some heterogeneity in parasite ages.

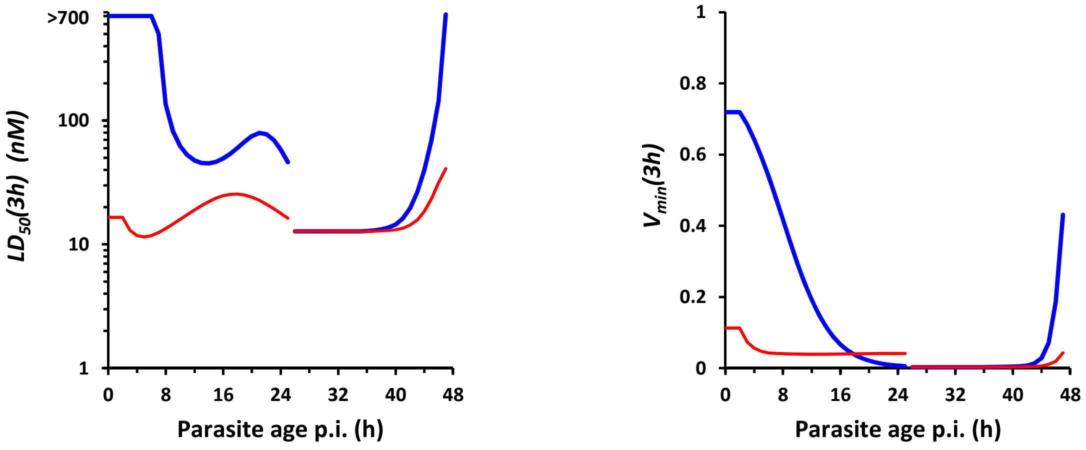

Figure SA3. Simulated variation of *LD_{50}(3h)* and *V_{min}(3h)* across the intraerythrocytic cycle. The age-dependent CED model parameters presented in Figure SA2 were used to calculate the *LD_{50}(3h)* and *V_{min}(3h)* values for the PL2 (red) and PL7 (blue) strains at different stages of the intraerythrocytic cycle. *LD_{50}* is the 50% lethal dose (*LD_{50}*) and *LD_{50}* is the minimum viability, *i.e.*, the viability at saturating drug concentrations.

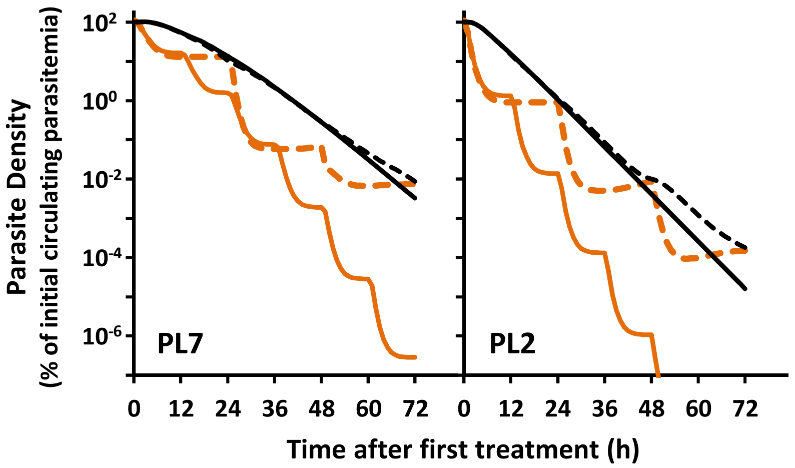

Figure SA4. Comparison of standard (dashed curves) and split-dose (solid curves) ART treatment regimens of PL2 and PL7-like infections. Orange curves represent the total number of viable parasites (*i.e.* sequestered + circulating) and black curves the parasite clearance curves (*i.e.* the total circulating parasites including viable and non-viable parasites). Simulations performed as described in text.

References

1. Klonis N, Xie SC, McCaw JM, Crespo-Ortiz MP, Zaloumis SG, et al. (2013) Altered temporal response of malaria parasites determines differential sensitivity to artemisinin. Proc Natl Acad Sci U S A 110: 5157-5162.
2. Saralamba S, Pan-Ngum W, Maude RJ, Lee SJ, Tarning J, et al. (2011) Intrahost modeling of artemisinin resistance in *Plasmodium falciparum*. Proc Natl Acad Sci U S A 108: 397-402.
3. Simpson JA, Zaloumis S, DeLivera AM, Price RN, McCaw JM (2014) Making the most of clinical data: reviewing the role of pharmacokinetic-pharmacodynamic models of anti-malarial drugs. AAPS J 16: 962-974.
4. Dondorp AM, Nosten F, Yi P, Das D, Phyo AP, et al. (2009) Artemisinin resistance in Plasmodium falciparum malaria. The New England journal of medicine 361: 455-467.
5. White NJ (2011) The parasite clearance curve. Malaria journal 10: 278.
6. Das D, Tripura R, Phyo AP, Lwin KM, Tarning J, et al. (2013) Effect of high-dose or split-dose artesunate on parasite clearance in artemisinin-resistant falciparum malaria. Clin Infect Dis 56: e48-58.
