# Supplementary figures and images for: Targeting the Cell Stress Response of Plasmodium falciparum to Overcome Artemisinin Resistance
Source: PLoS Biol. 2015 Apr 22;13(4):e1002132. doi: 10.1371/journal.pbio.1002132 (PMC4406523; doi:10.1371/journal.pbio.1002132)

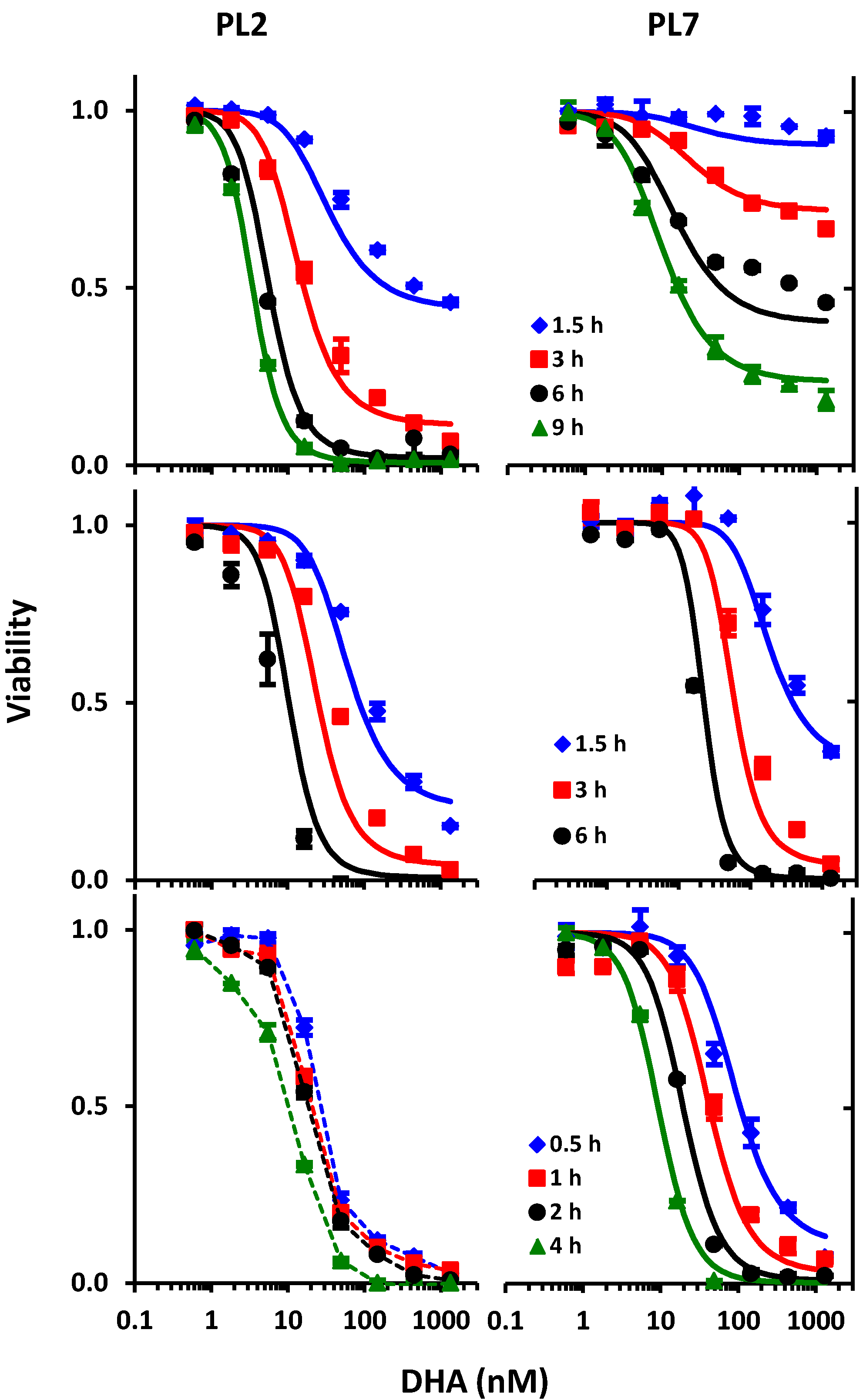

Supplement: S1 Fig — Measurements were carried out as described in Fig 1C, by exposing parasites of defined ages to DHA for the time periods indicated in the legends. Viability is defined as the fraction of the parasite population that survives drug exposure and is able to enter the next parasite cycle. The parasites were early rings (2 h p.i., top panel), rings (7 h p.i., middle), or trophozoites (34 h p.i., bottom). Solid curves represent the best fits with the CED model parameters shown in Table 1. Reliable parameters could not be obtained with the set of measurements represented by the dashed line because the drug dose was already saturating at the shortest exposure time (30 min). Error bars correspond to the range of duplicates. (TIFF) [file pbio.1002132.s003.tiff]

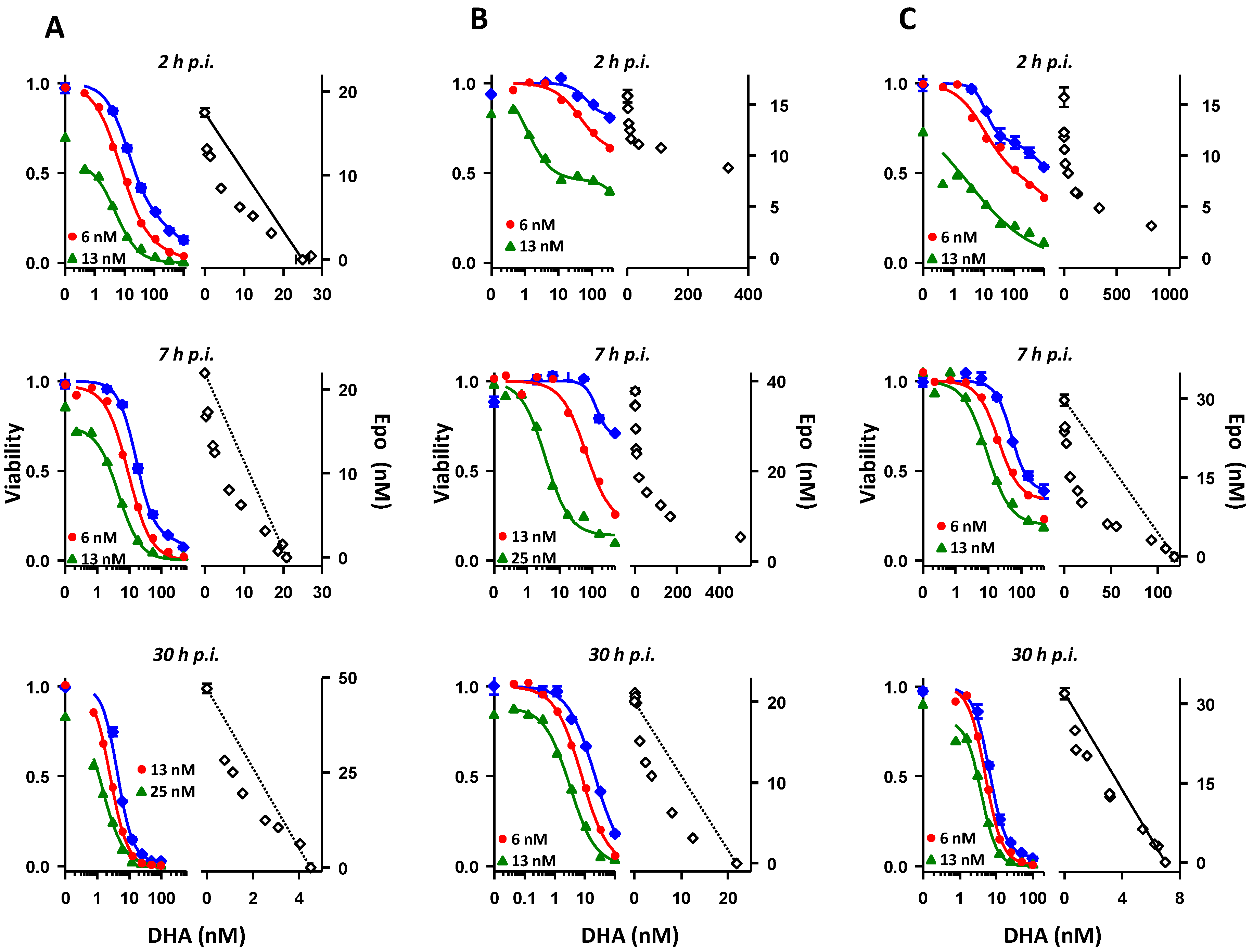

Supplement: S2 Fig — PL2 (A), PL5 (B), and PL7 (C) parasites at different stages (indicated by their p.i. age) were subjected to 3-h pulses in the presence of different combinations of DHA and epoxomicin (Epo). The left side of each panel shows the influence of sublethal concentrations of epoxomicin (indicated in figure) on the dose-response profile of DHA (blue symbols). The right side of each panel presents the isobolograms for the epoxomicin-DHA pair at the 50% LD 50 level. The dashed line is plotted between the LD 50 of each drug used alone, emphasizing the concave nature of many of the isobolograms. The absence of a line indicates the LD 50 value of one of the drugs was outside the range of concentrations examined. Error bars, where present, correspond to the range of duplicates. (TIFF) [file pbio.1002132.s004.tiff]

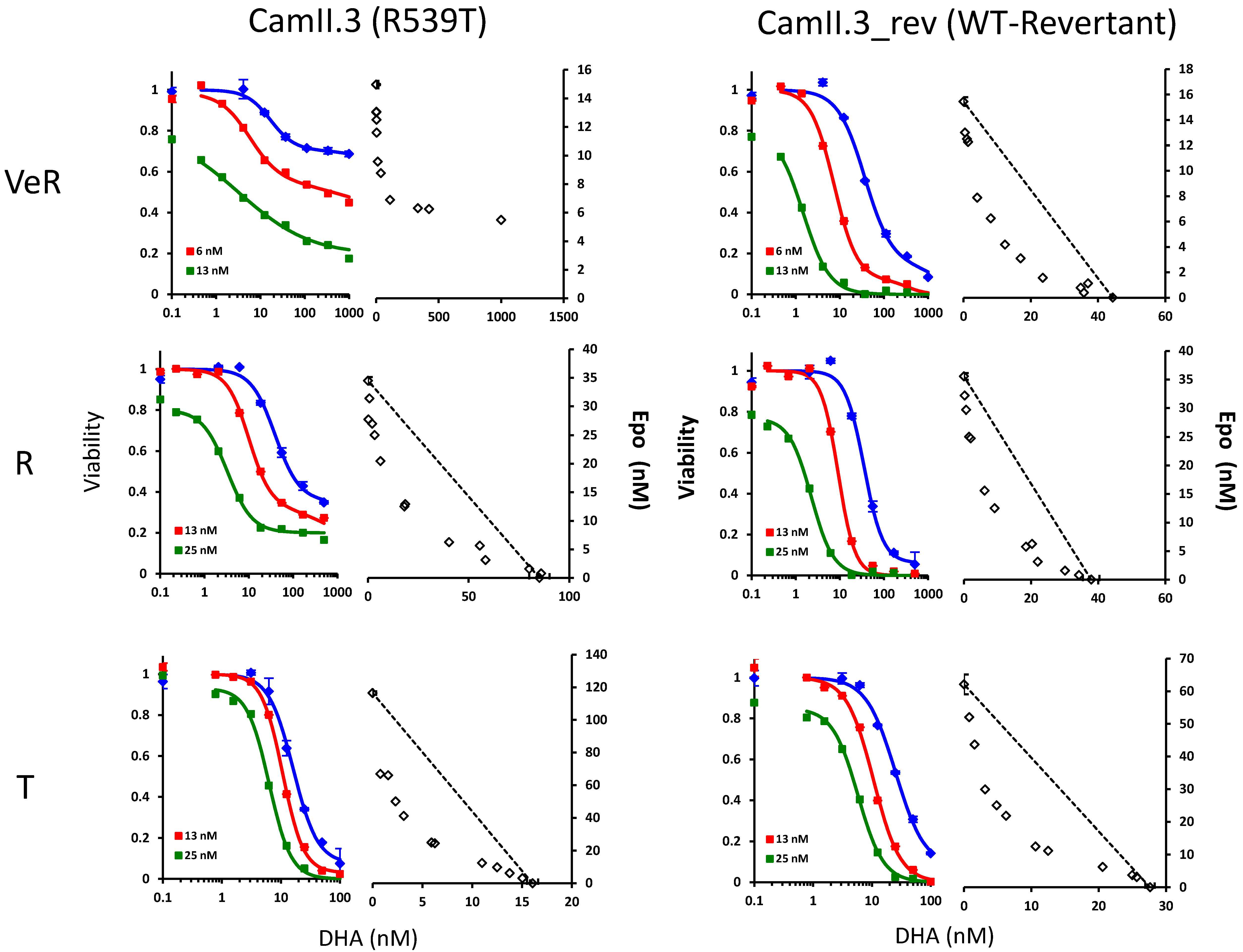

Supplement: S3 Fig — Cam3.II (R539T) (A) and a reverted transfectant in which the K13 wild-type genotype has been restored (Cam3.II_rev) (B) were synchronized to a 1-h window and subjected to 3-h pulses in the presence of different combinations of DHA and epoxomicin (Epo) at the following times p.i.: 1 h (very early ring, vER), 7 h (ring, R), and 27 h (troph, T). The left panels show the dose-responses to DHA and the influence of sub-lethal concentrations (indicated) of epoxomicin. The right panels show the isobolograms for the epoxomicin-DHA pairs at the 50% lethal dose level. Error bars, where present, correspond to the range of duplicates. (TIFF) [file pbio.1002132.s005.tiff]

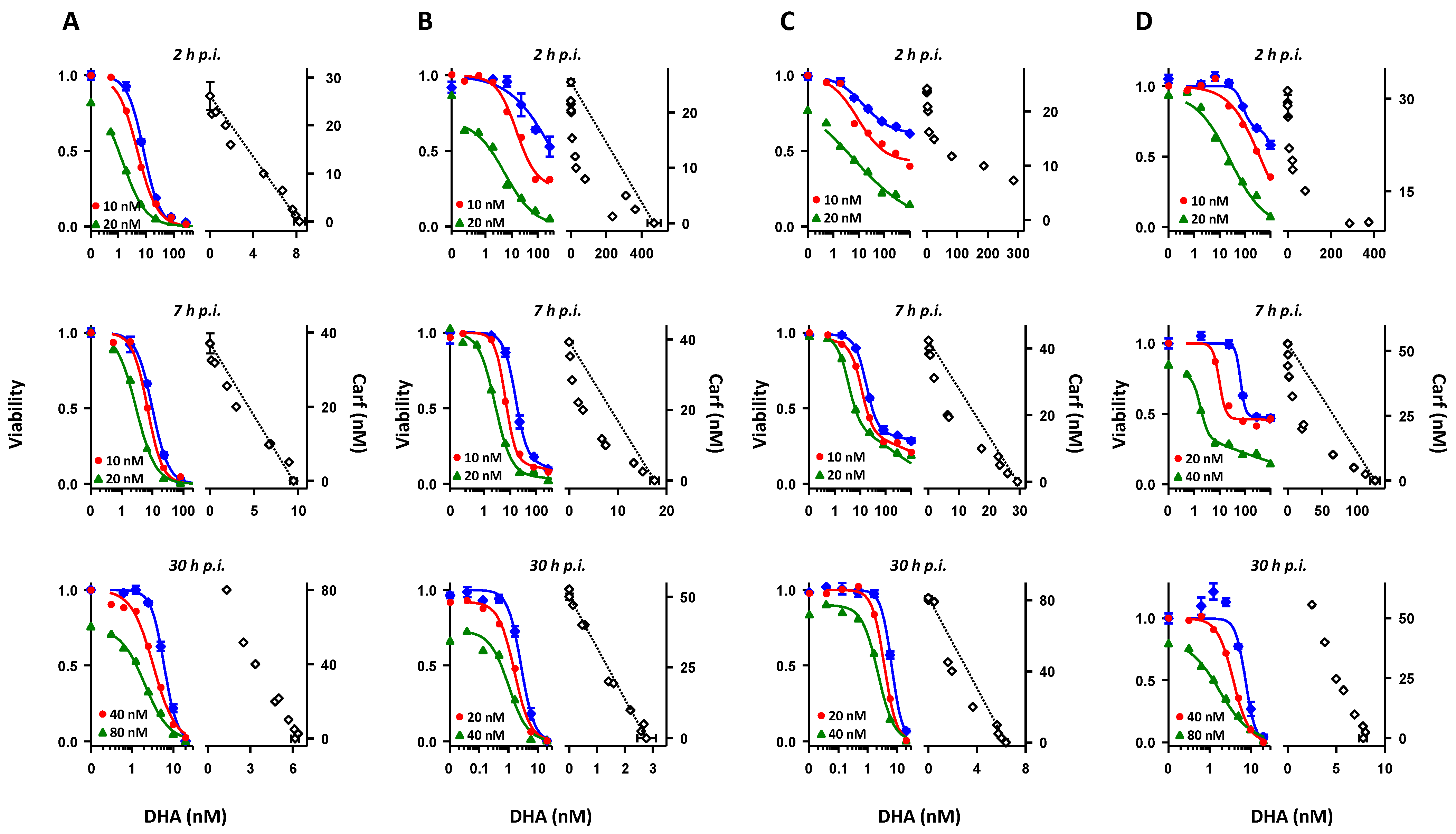

Supplement: S4 Fig — PL2 (A), PL1 (B), PL7 (C), and PL5 (D) parasites at different stages (indicated by their p.i. age) were subjected to 3-h pulses in the presence of different combinations of DHA and carfilzomib (Carf). The left side of each panel shows the influence of sublethal concentrations of carfilzomib (indicated in figure) on the dose-response profile of DHA (blue symbols). The right side of each panel presents the isobolograms for the carfilzomib-DHA pair at the 50% LD 50 (3h) level. The dashed line is plotted between the LD 50 (3h) of each drug used alone, emphasizing the concave nature of many of the isobolograms. The absence of a line indicates the LD 50 (3h) value of one of the drugs was outside the range of concentrations examined. Error bars, where present, correspond to the range of duplicates from an experiment performed in singlicate. (TIFF) [file pbio.1002132.s006.tiff]

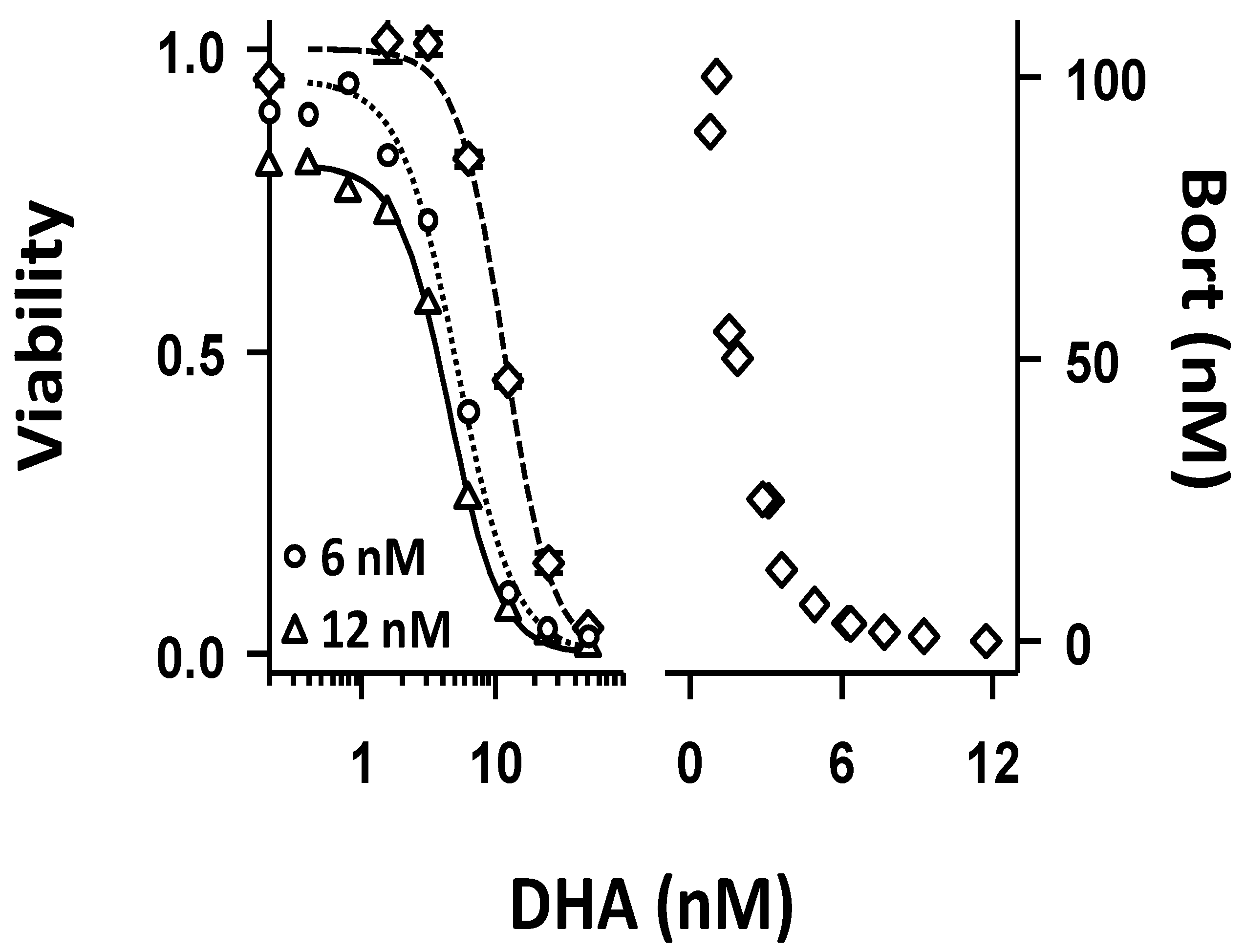

Supplement: S5 Fig — PL7 parasites (3-h window, mid-trophozoites) were subjected to 3-h pulses in the presence of different combinations of DHA and Bortezomib (Bort). The left panel shows the dose-responses to DHA (diamonds) and the influence of sub-lethal concentrations (indicated) of Bortezomib. The right panel shows the isobologram for the Bortezomib-DHA pair at the 50% lethal dose level. Error bars, where present, correspond to the range of duplicates. (TIFF) [file pbio.1002132.s007.tiff]

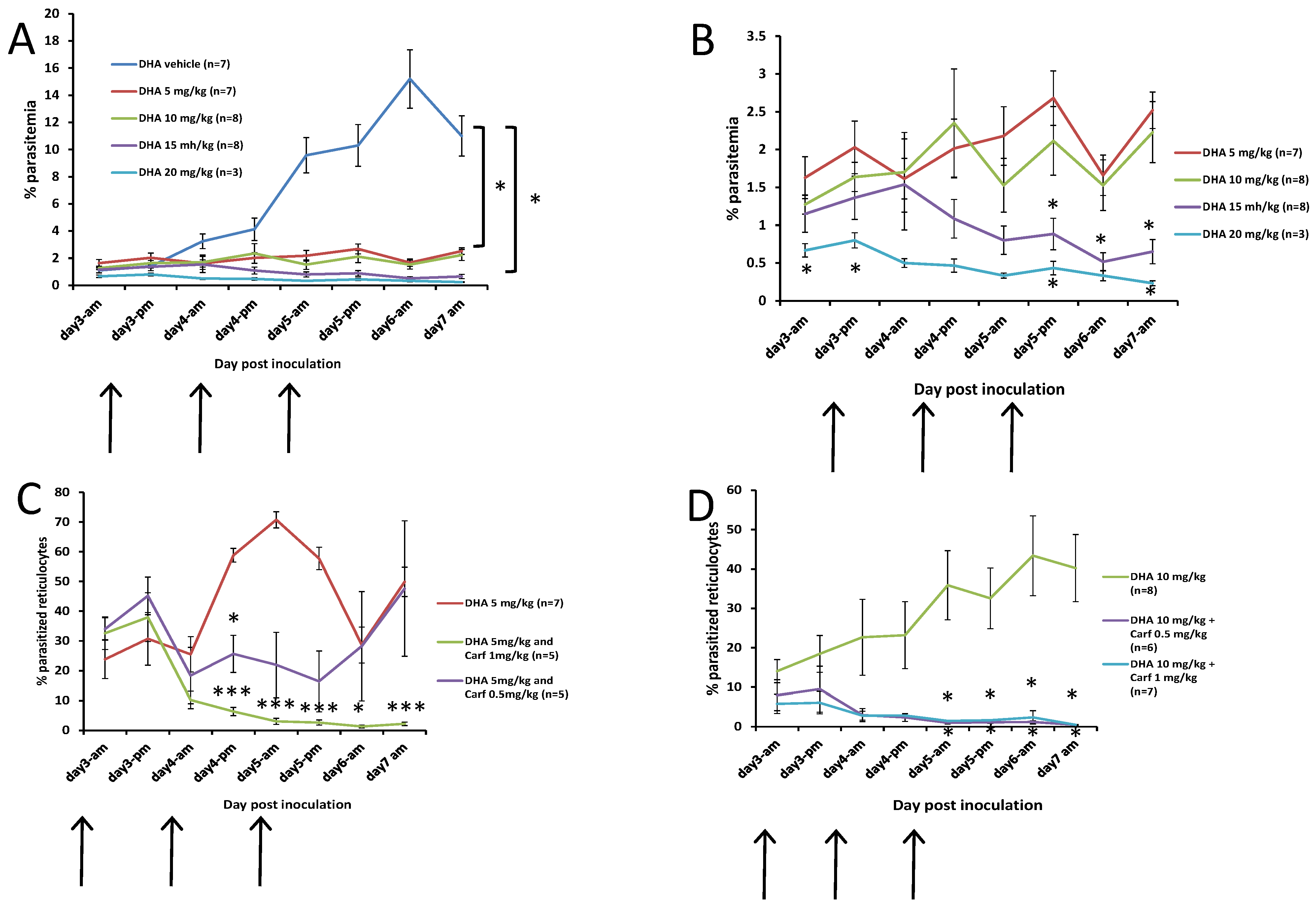

Supplement: S6 Fig — Balb/c mice were infected with 106 parasites (P. berghei) and treatment was initiated at ~1% parasitaemia (i.e., day 3), indicated with an arrow. (A) Delivery of vehicle alone is associated with high parasitemia (in RBCs), while DHA alone has a significant effect on parasite burden at all doses. (B) The same data displayed on an expanded scale reveals that drug treatment with DHA at doses 5 and 10 mg/kg has a moderate effect on parasite growth, whereas the doses 15 and 20 mg/kg totally abrogate parasite growth. (C) DHA (5 mg/kg) + Carfilzomib (0.5 mg/kg) modestly prevents parasite growth in reticulocytes, whereas DHA (5 mg/kg) + Carfilzomib (1 mg/kg) drastically reduces the parasite burden after treatment. (C) DHA (10 mg/kg) + Carfilzomib (0.5 or 1 mg/kg) abrogate the growth of P. berghei parasites in reticulocytes. Error bars represent SEM, and * and *** represent, respectively, p < 0.05 and p < 0.01 after Bonferroni correction for multiple testing. (TIFF) [file pbio.1002132.s008.tiff]

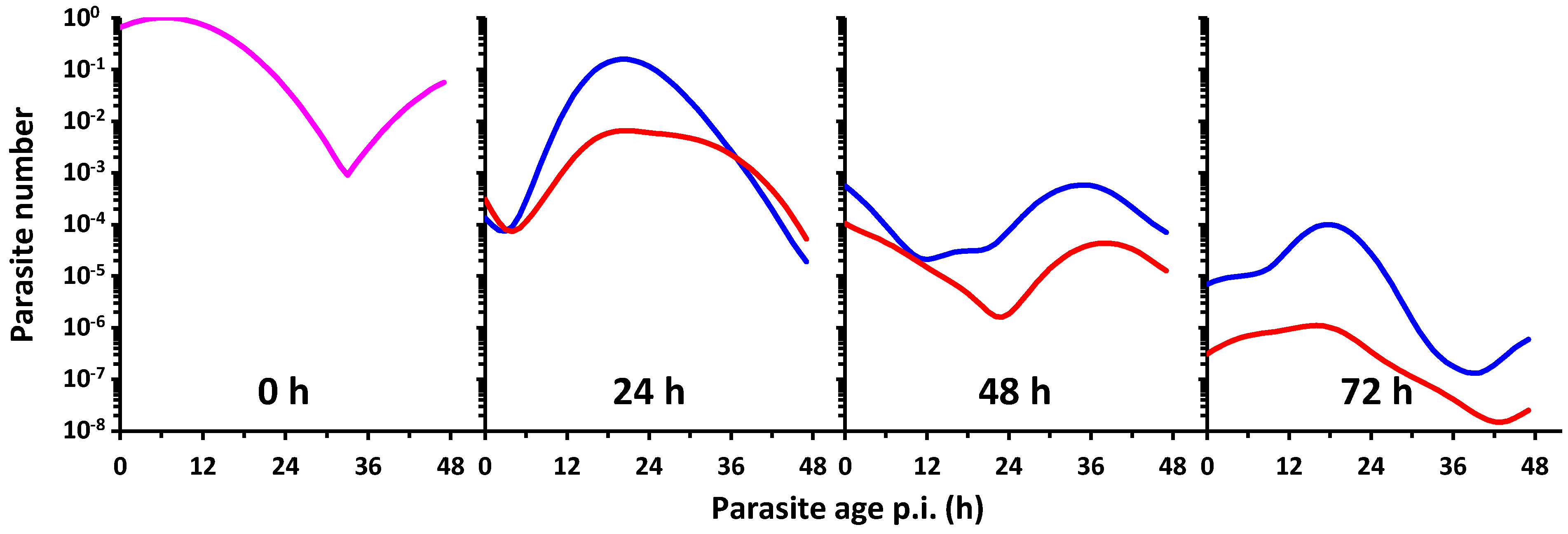

Supplement: S7 Fig — (TIFF) [file pbio.1002132.s009.tiff]

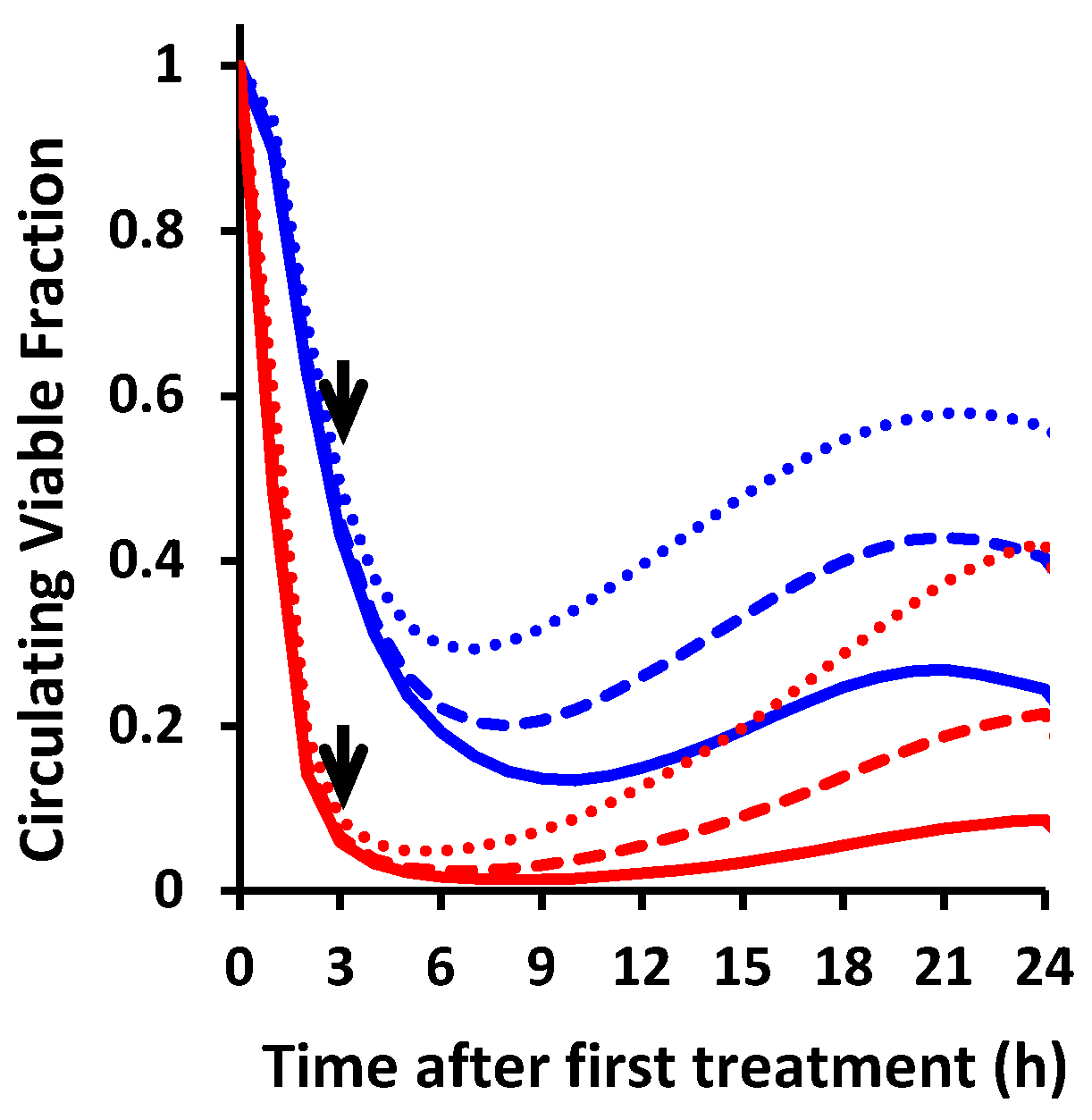

Supplement: S8 Fig — Simulations for PL2 (red) and PL7-like (blue) infections were performed as described in Fig 7 but using various values for C max (0.5, 2, and 20 μM corresponding to dotted, dashed, and solid lines, respectively). The circulating viable fraction at each time is the total number of circulating viable parasites divided by the total number of circulating parasites (i.e., including unviable parasites). Arrows indicate that this parameter at 3 h is insensitive to the value of C max and can distinguish between the two strains. (TIFF) [file pbio.1002132.s010.tiff]
