# Supplementary material for: Targeting the Cell Stress Response of Plasmodium falciparum to Overcome Artemisinin Resistance
Source: PLoS Biol. 2015 Apr 22;13(4):e1002132. doi: 10.1371/journal.pbio.1002132 (PMC4406523; doi:10.1371/journal.pbio.1002132)
Supplement: S1 Table — (PDF) [file pbio.1002132.s012.pdf]

**Table S1. Quality of Illumina MiSeq genome sequence data.**

| Strain | Total sequence bases | Number of paired reads (2x) | Number of mapped reads (1x) | Percentage of reference sequenced | Percentage of reference with $\geq$ 5 reads | Mean coverage ± SD | Mean mapping quality |
|---|---|---|---|---|---|---|---|
| PL1 | 1,695,850,781 | 6,253,736 | 3,165,344 | 96.17% | 94.07% | 73 ± 40 | 58.22 |
| PL2 | 1,840,022,251 | 6,843,040 | 3,462,925 | 96.37% | 93.92% | 79 ± 46 | 58.13 |
| PL5 | 1,898,391,977 | 7,052,785 | 3,572,673 | 95.73% | 94.64% | 82 ± 39 | 58.10 |
| PL7 | 1,811,579,470 | 6,710,022 | 3,393,759 | 95.85% | 94.37% | 78 ± 38 | 58.14 |
