# Supplementary material for: Targeting the Cell Stress Response of Plasmodium falciparum to Overcome Artemisinin Resistance
Source: PLoS Biol. 2015 Apr 22;13(4):e1002132. doi: 10.1371/journal.pbio.1002132 (PMC4406523; doi:10.1371/journal.pbio.1002132)
Supplement: S2 Table — Kelch 13 (K13), P. falciparum Multi-Drug Resistance Gene-2 (MDR2), apicoplast ribosomal protein (ARPS10), ferredoxin (FD), PfCRT, protein phosphatase (PPH), phosphoinositide-binding protein (PIB7), Multi-Drug Resistance Gene-1 (MDR1), Ubiquitin Binding Protein-1 (UBP1), Multidrug Resistance Protein-1 (MRP1), cyclic nucleotide-binding protein (cNBP) and the 3′ UTR of the DNA polymerase catalytic subunit. No artemisinin resistance associated mutations were observed in RAD5 or SERCA. (PDF) [file pbio.1002132.s013.pdf]

Table S2. SNP analysis of Pailin strains at loci reported to be associated with differences in ART sensitivity.

Kelch 13 (K13), *P. falciparum* Multi-Drug Resistance Gene-2 (MDR2), apicoplast ribosomal protein (ARPS10), ferredoxin (FD), PfCRT, protein phosphatase (PPH), phosphoinositide-binding protein (PIB7), Multi-Drug Resistance Gene-1 (MDR1), Ubiquitin Binding Protein-1 (UBP1), Multidrug Resistance Protein-1 (MRP1), cyclic nucleotide-binding protein (cNBP) and the 3' UTR of the DNA polymerase catalytic subunit. No artemisinin resistance associated mutations were observed in RAD5 or SERCA.

**Gene**	**PlasmoDB ID**	**NT**	**SNP type**	**Codon**	**3D7ref**	**PL1**	**PL2**	**PL5**	**PL7**
**K13**	PF3D7_1343700	1725259	NSY	C580Y	C	C	C	T	C
		1725382	NSY	R539T	C	C	C	C	G
		1725521	NSY	Y493H	A	G	A	A	A
**MDR2**	PF3D7_1447900	1956225	NSY	T484I	G	A	G	A	A
**ARPS10**	PF3D7_1460900.1	2481070	NSY	V127M	G	A	G	A	A
**FD**	PF3D7_1318100	748395	NSY	D193Y	C	A	A	A	A
**PfCRT**	PF3D7_0709000	405362	NSY	N326S	A	G	G	G	G
		405600	NSY	I356T	T	C	T	C	C
**PPH**	PF3D7_1012700	490720	NSY	V1157L	G	G	C	C	C
**PIB7**	PF3D7_0720700	405362	NSY	C1484F	G	G	G	T	T
**MDR1**	PF3D7_0523000	405600	NSY	N86Y	A	A	T	A	A
**UBP1**	PF3D7_0104300 / PFA0220w	490720	NSY	E1528D	A	A	A	C	A
**MRP1**	PF3D7_0112200	896660	NSY	I876V	A	G	G	G	A
**NBP**	PF3D7_1417400	958145	NSY	S993L	C	T	C	T	T
**DNApol**	PF3D7_1017000	194852	3' UTR	95	T	A	A	A	T

Legend:

SYN SYNONYMOUS

NSY NON-SYNONYMOUS

Codon — Codon containing a SNP reported to be associated with ART resistance ([17], [26], [18]

HOMOZYGOUS_REFERENCE

HOMOZYGOUS_ALTERNATE
