# Supplementary material for: Targeting the Cell Stress Response of Plasmodium falciparum to Overcome Artemisinin Resistance
Source: PLoS Biol. 2015 Apr 22;13(4):e1002132. doi: 10.1371/journal.pbio.1002132 (PMC4406523; doi:10.1371/journal.pbio.1002132)
Supplement: S3 Table — (PDF) [file pbio.1002132.s014.pdf]

Table S3. Parameters used in simulation presented in Figure 7A*

Parameter	Value	Comments
DHA Pharmacokinetic Parameters		
C_{max}	2820 nM	Maximum *in vivo* concentration
Time to C_{max}	1 h	Time to reach C_{max} (linear)
Elimination half-life	51 min	
Initial Parasite Age Distribution		
μ_{age}	7 h	Average parasite age
σ_{age}	7 h	Standard deviation of age distribution
Age Distribution Broadening		
σ_{broad}	3 h	Standard deviation per 24 h period
Parasite Replication		
Amplification	10 per cycle	
Sequestration Parameters		
a_b	15 h	Parasite age at which sequestration begins
a_{50}	18 h	Parasite age at which 50% are sequestered
Growth Retardation Parameters		
Growth delay	6 h	
Time over which delay occurs	12 h	

* See S1 Appendix for detailed description of parameters
